# Supplementary material for: Accurate phenotypic classification and exome sequencing allow identification of novel genes and variants associated with adult-onset hearing loss
Source: medRxiv. 2023 Apr 29:2023.04.27.23289040. Preprint. [Version 1] doi: 10.1101/2023.04.27.23289040 (PMC10168485; doi:10.1101/2023.04.27.23289040)
Supplement: Supplement 1 [file media-1.pdf]

Supplementary Figures

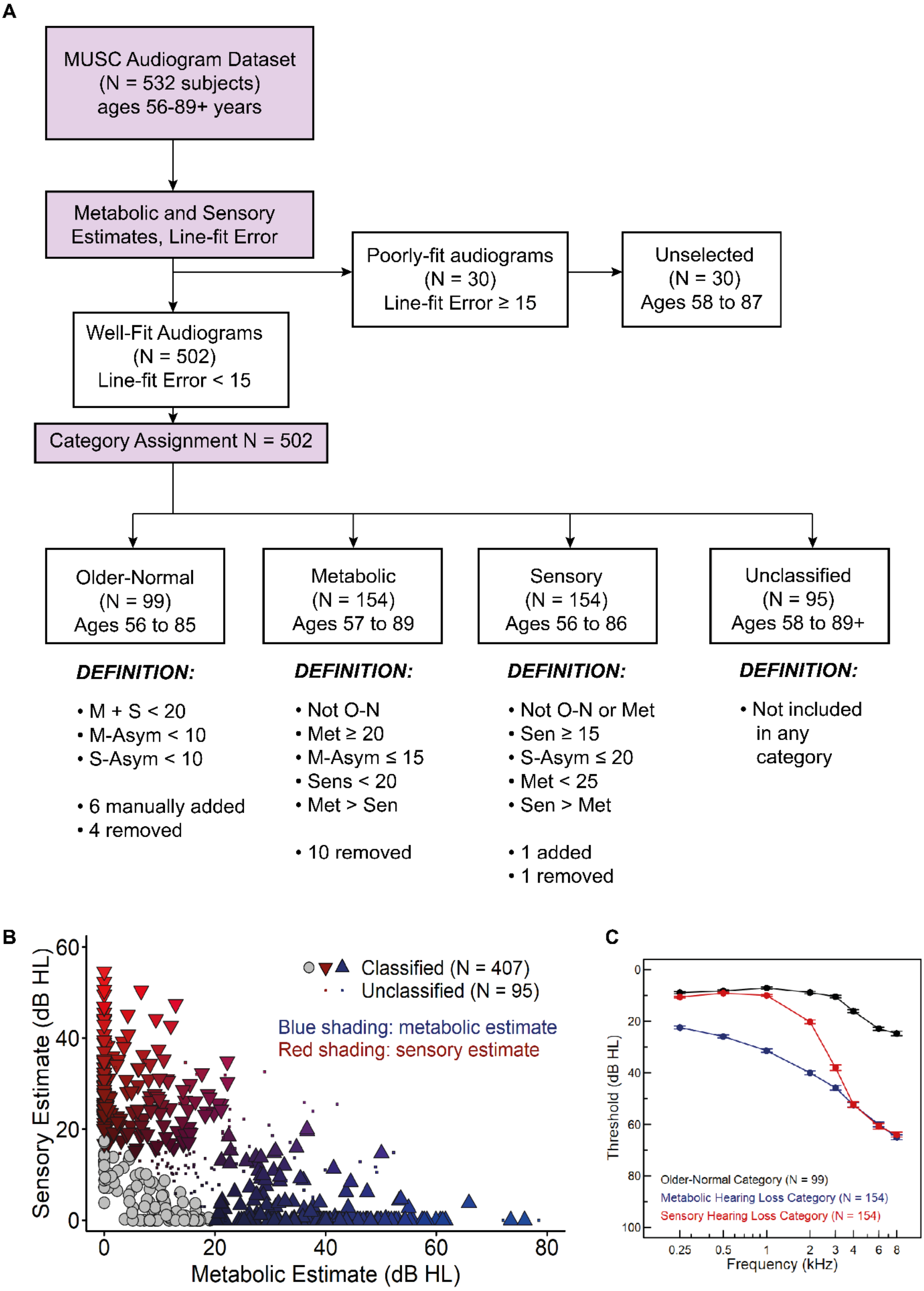

Suppl. Figure 1. A, Flow chart showing the audiogram classification procedure for the MUSC cohort. B, plot showing the sensory estimate (how much of the hearing loss observed can be attributed to sensory hearing loss) against the metabolic estimate (how much of the hearing loss observed can be attributed to metabolic hearing loss) for each case, with the blue/red shading indicating the magnitude of each estimate (metabolic/sensory respectively). The small dots are the Unclassified cases; Unselected cases are not shown. The grey circles near the origin are the Older-Normal participants, with very small contributions of both Sensory and Metabolic hearing loss. The red-shaded triangles aligned along the Y axis are those people with purely sensory contributions to their hearing loss, and the blue-shaded triangles along the X axis are those people with purely metabolic contributions to their hearing loss. However, there are a substantial number of people with mixed contributions, and these are the mixed shades in the middle of the graph. C, mean audiograms for the selected cases assigned to each category. Error bars are SEM.

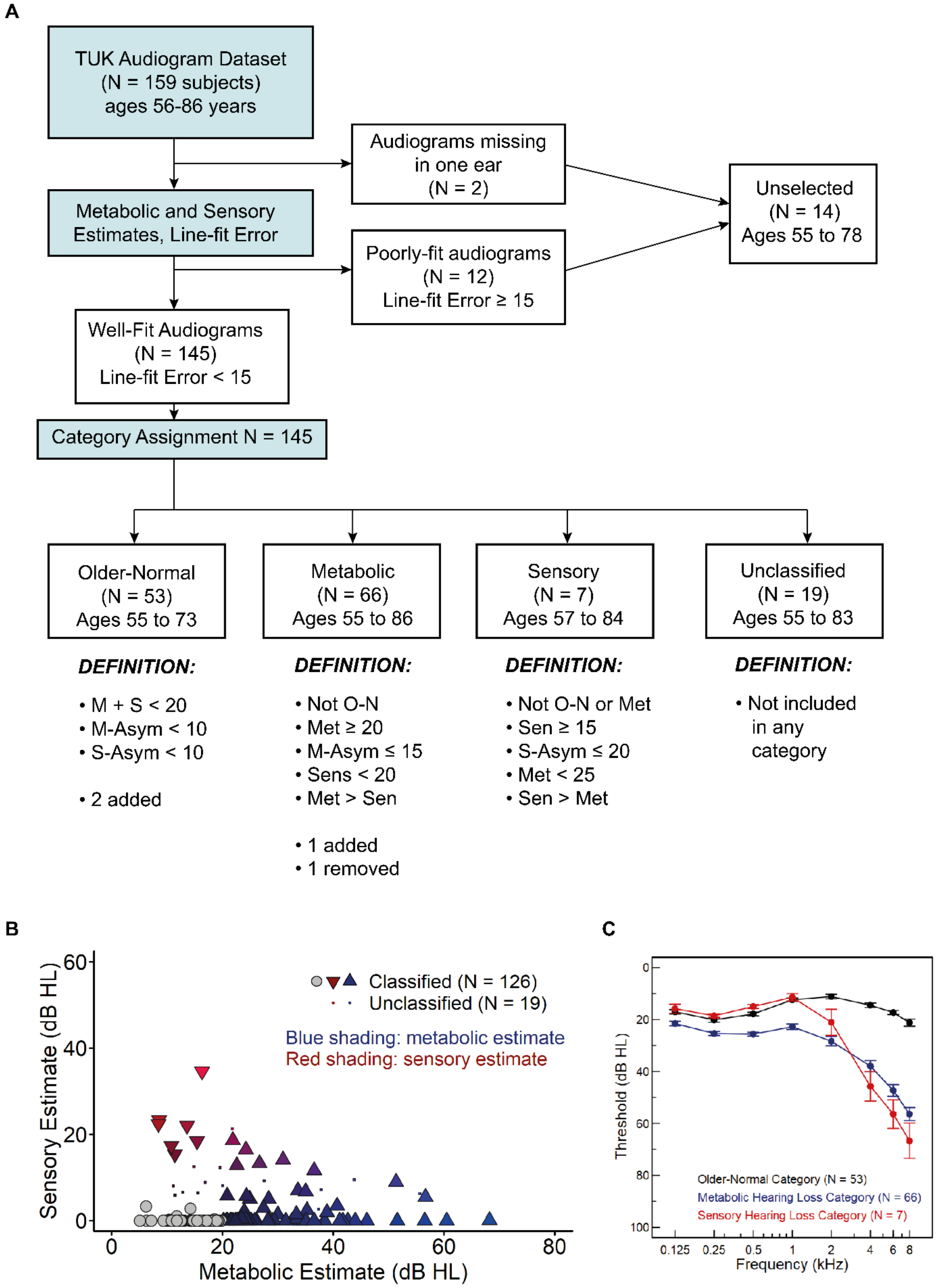
A
TUK Audiogram Dataset (N = 159 subjects) ages 56-86 years
Audiograms missing in one ear (N = 2)
Unselected (N = 14) Ages 55 to 78
Metabolic and Sensory Estimates, Line-fit Error
Poorly-fit audiograms (N = 12) Line-fit Error ≥ 15
Well-Fit Audiograms (N = 145) Line-fit Error < 15
Category Assignment N = 145
Older-Normal (N = 53) Ages 55 to 73
Metabolic (N = 66) Ages 55 to 86
Sensory (N = 7) Ages 57 to 84
Unclassified (N = 19) Ages 55 to 83
DEFINITION:
• M + S < 20
• M-Asym < 10
• S-Asym < 10
• 2 added
DEFINITION:
• Not O-N
• Met ≥ 20
• M-Asym ≤ 15
• Sens < 20
• Met > Sen
• 1 added
• 1 removed
DEFINITION:
• Not O-N or Met
• Sen ≥ 15
• S-Asym ≤ 20
• Met < 25
• Sen > Met
DEFINITION:
• Not included in any category
B
Sensory Estimate (dB HL)
Metabolic Estimate (dB HL)
Classified (N = 126)
Unclassified (N = 19)
Blue shading: metabolic estimate
Red shading: sensory estimate
0
20
40
60
80
C
Threshold (dB HL)
Frequency (kHz)
0.125
0.25
0.5
1
2
4
6
8
100
Older-Normal Category (N = 53)
Metabolic Hearing Loss Category (N = 66)
Sensory Hearing Loss Category (N = 7)

Suppl. Figure 2. A, Flow chart showing the audiogram classification procedure for the TwinsUK cohort. B, plot showing the sensory estimate (how much of the hearing loss observed can be attributed to sensory hearing loss) against the metabolic estimate (how much of the hearing loss observed can be attributed to metabolic hearing loss) for each case, with the blue/red shading indicating the magnitude of each estimate (metabolic/sensory respectively). The small dots are the Unclassified cases; Unselected cases are not shown. The grey circles near the origin are the Older-Normal participants, with very small contributions of both Sensory and Metabolic hearing loss. The blue-shaded triangles along the X axis are those people with purely metabolic contributions to their hearing loss. There are no participants with a purely sensory contribution to their hearing loss, but there are a few with a Sensory classification, shown by redder shading and their high y coordinate. Most people with mixed contributions have a comparatively small Sensory component, and are classified as Metabolic (the blue-purple triangles on the right of the plot). C, mean audiograms for the selected cases assigned to each category. Error bars are SEM.

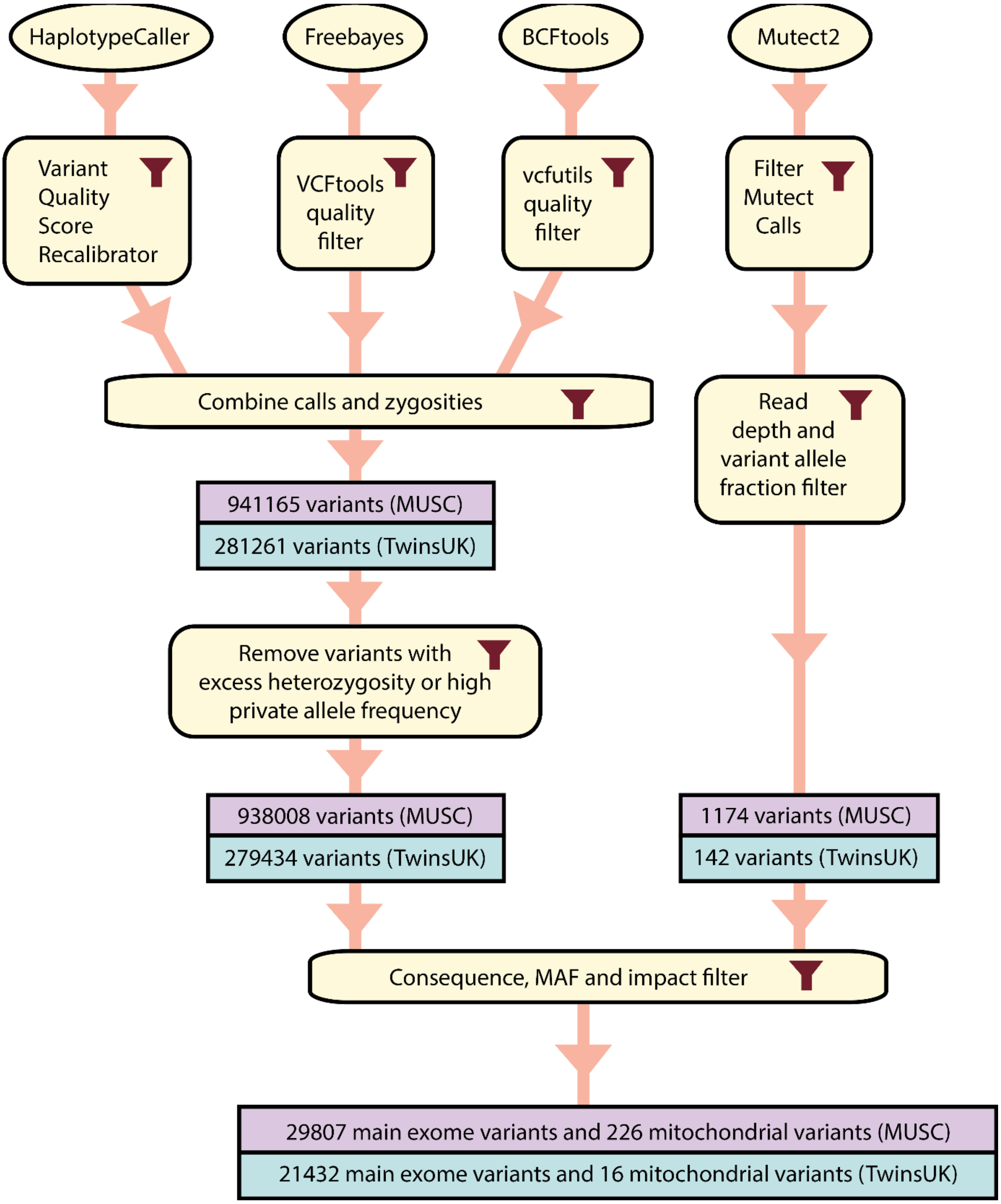

Suppl. Figure 3. Flow chart showing the variant filtering pipeline. See Suppl. Table 2 for details of the filter settings.

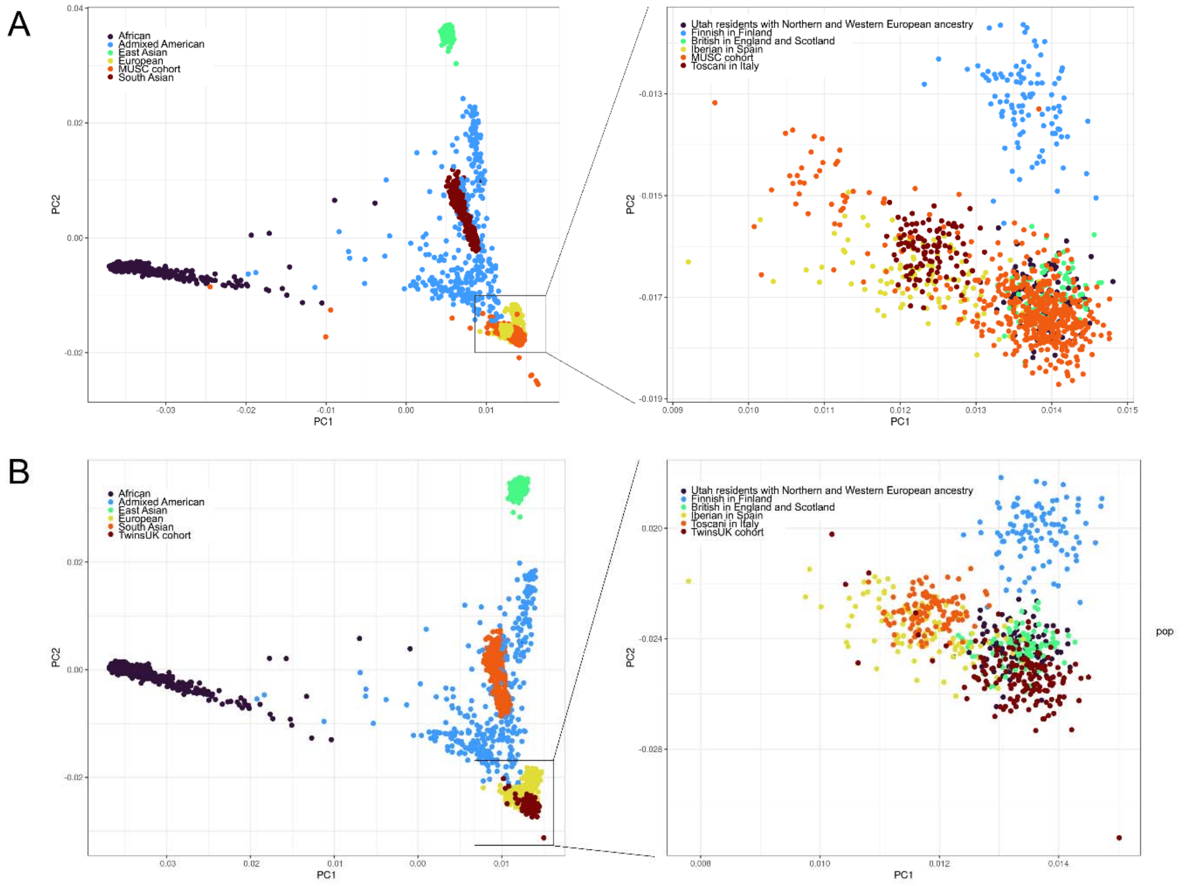

Suppl. Figure 4. Principal Component Analysis plots showing the genetic ancestry of the two cohorts used in this study compared to 2504 individuals from the 1000 Genomes study (Genomes Project et al. 2015). A shows the MUSC cohort, and B the TwinsUK cohort. On the left-hand plot, 1000 Genomes samples are coloured by their superpopulation, while the right-hand plot is a close-up showing just the European subpopulations.

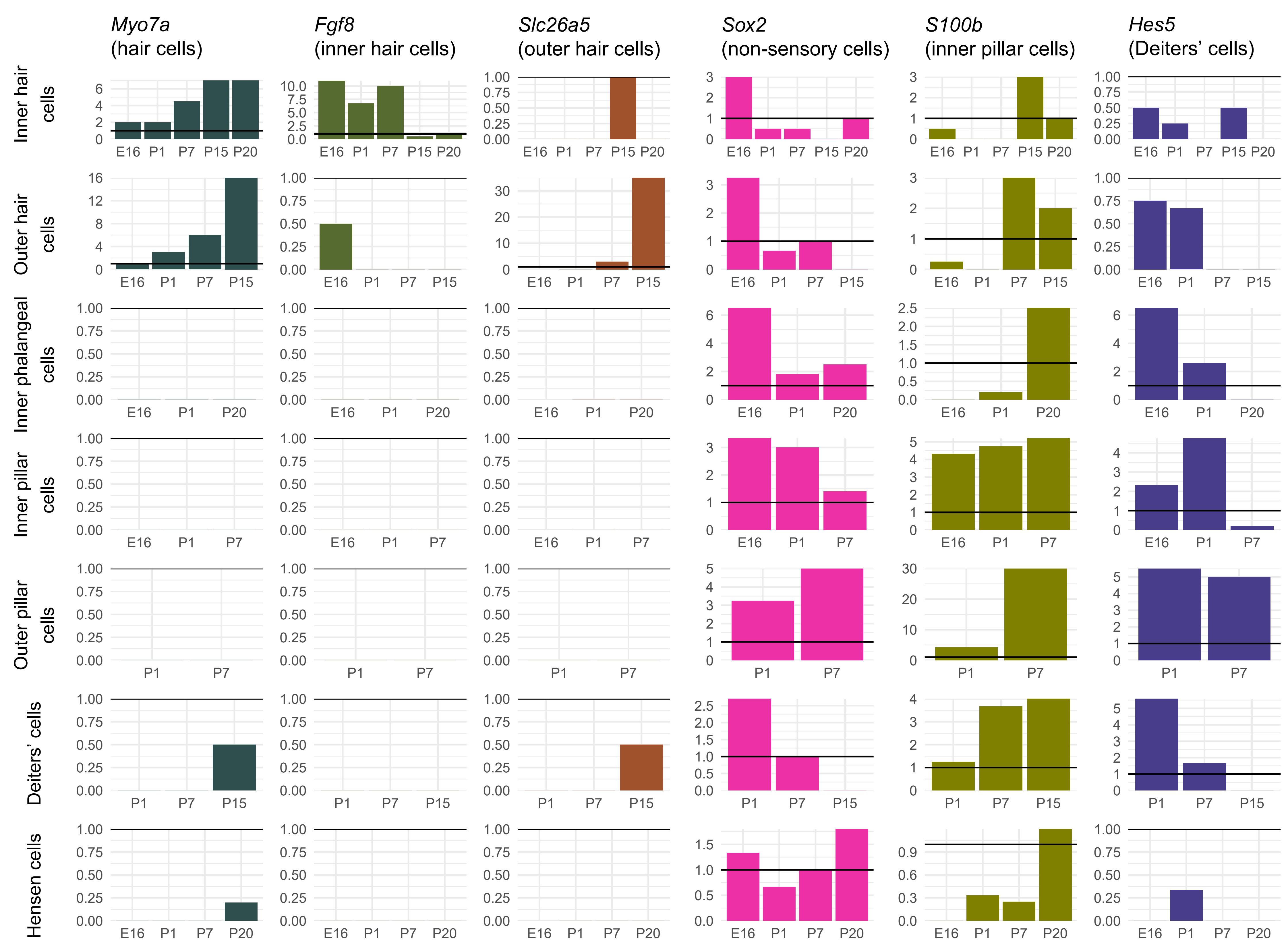

Myo7a (hair cells)
Fgf8 (inner hair cells)
Slc26a5 (outer hair cells)
Sox2 (non-sensory cells)
S100b (inner pillar cells)
Hes5 (Deiters' cells)
Inner hair cells
Outer hair cells
Inner phalangeal cells
Inner pillar cells
Outer pillar cells
Deiters' cells
Hensen cells
E16
P1
P7
P15
P20

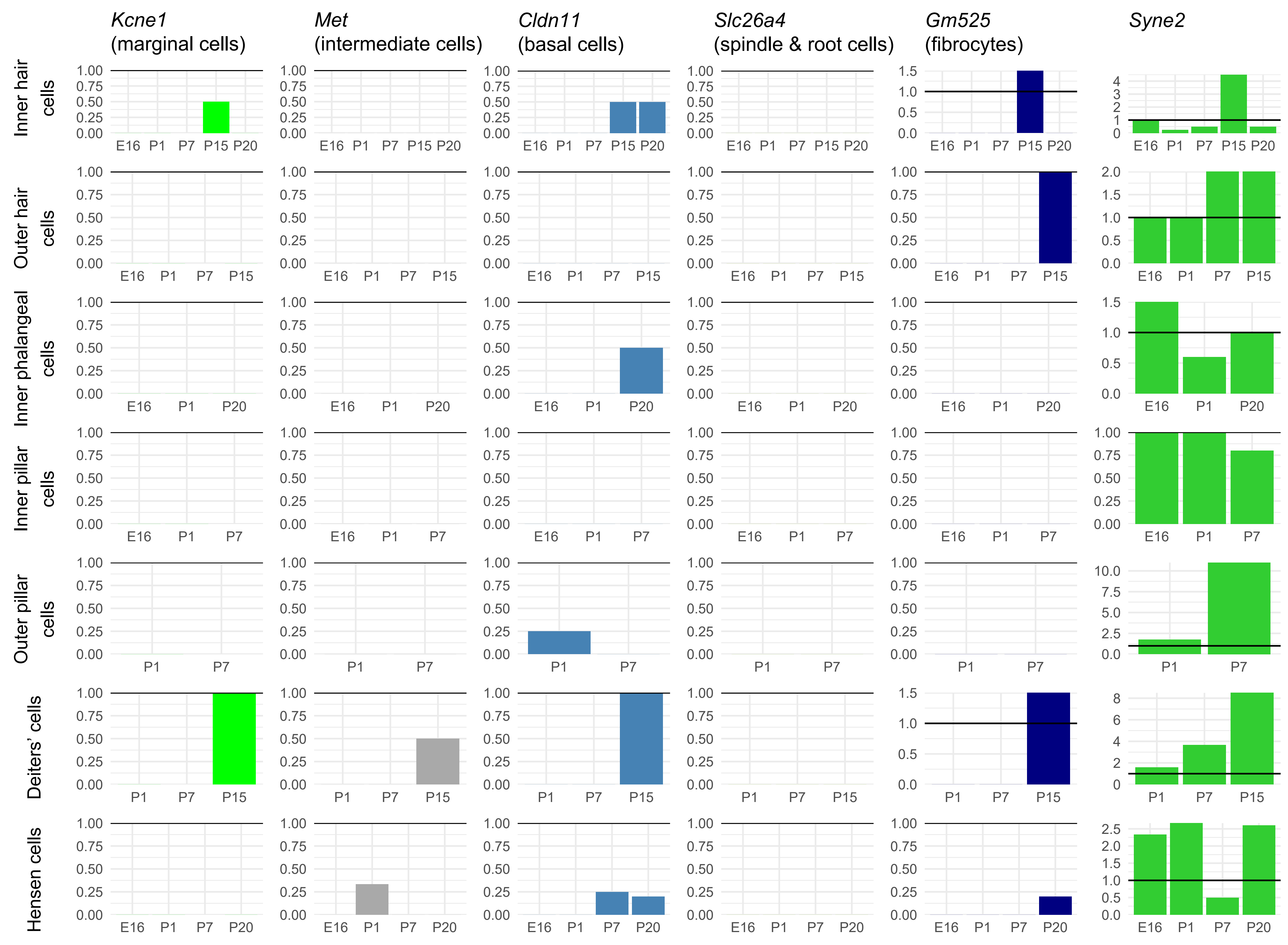

Kcne1
(marginal cells)
Met
(intermediate cells)
Cldn11
(basal cells)
Slc26a4
(spindle & root cells)
Gm525
(fibrocytes)
Syne2
Inner hair cells
Outer hair cells
Inner phalangeal cells
Inner pillar cells
Outer pillar cells
Deiters' cells
Hensen cells
E16
P1
P7
P15
P20

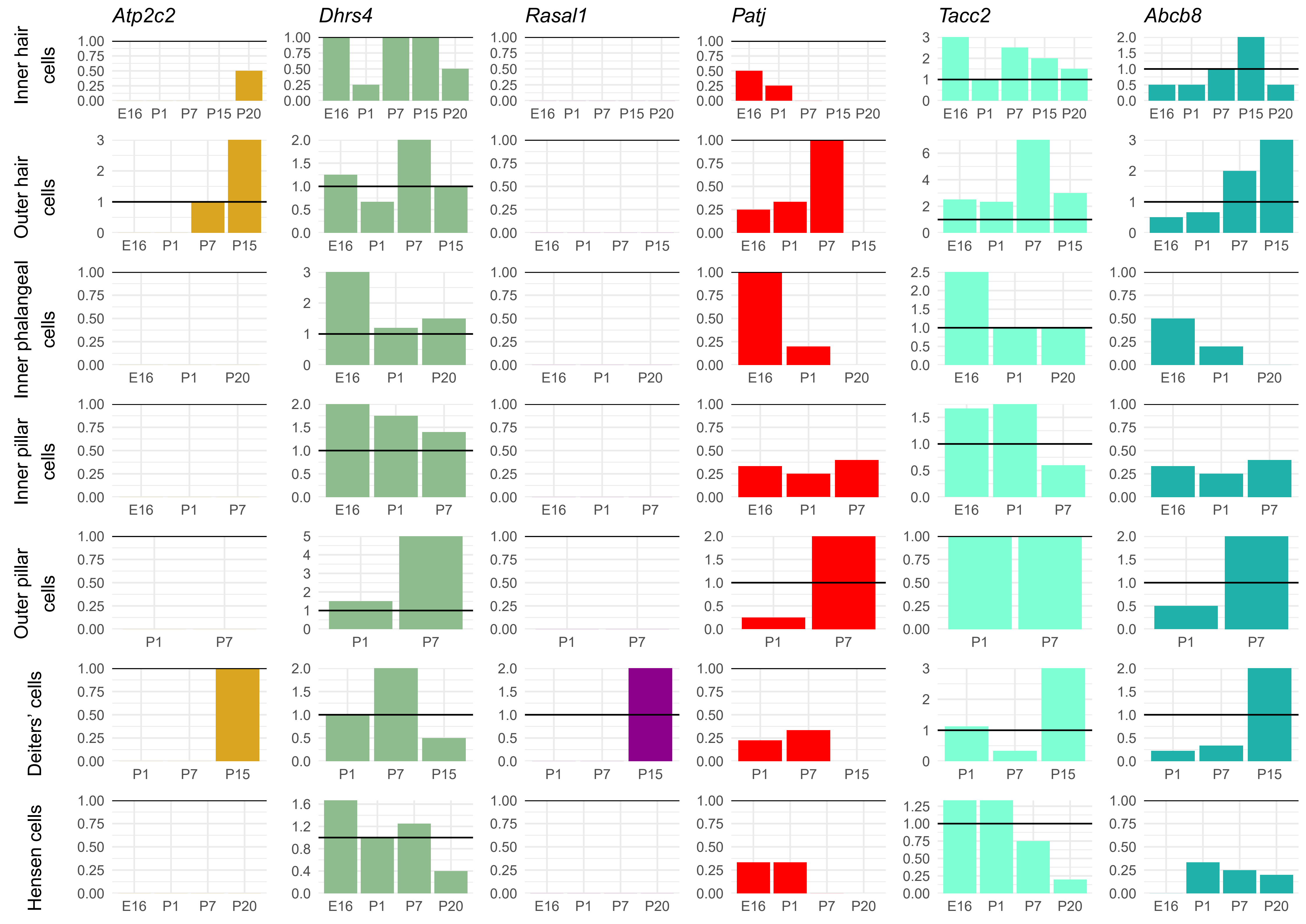

Atp2c2
Dhrs4
Rasal1
Patj
Tacc2
Abcb8
Inner hair cells
Outer hair cells
Inner phalangeal cells
Inner pillar cells
Outer pillar cells
Deiters' cells
Hensen cells
E16
P1
P7
P15
P20

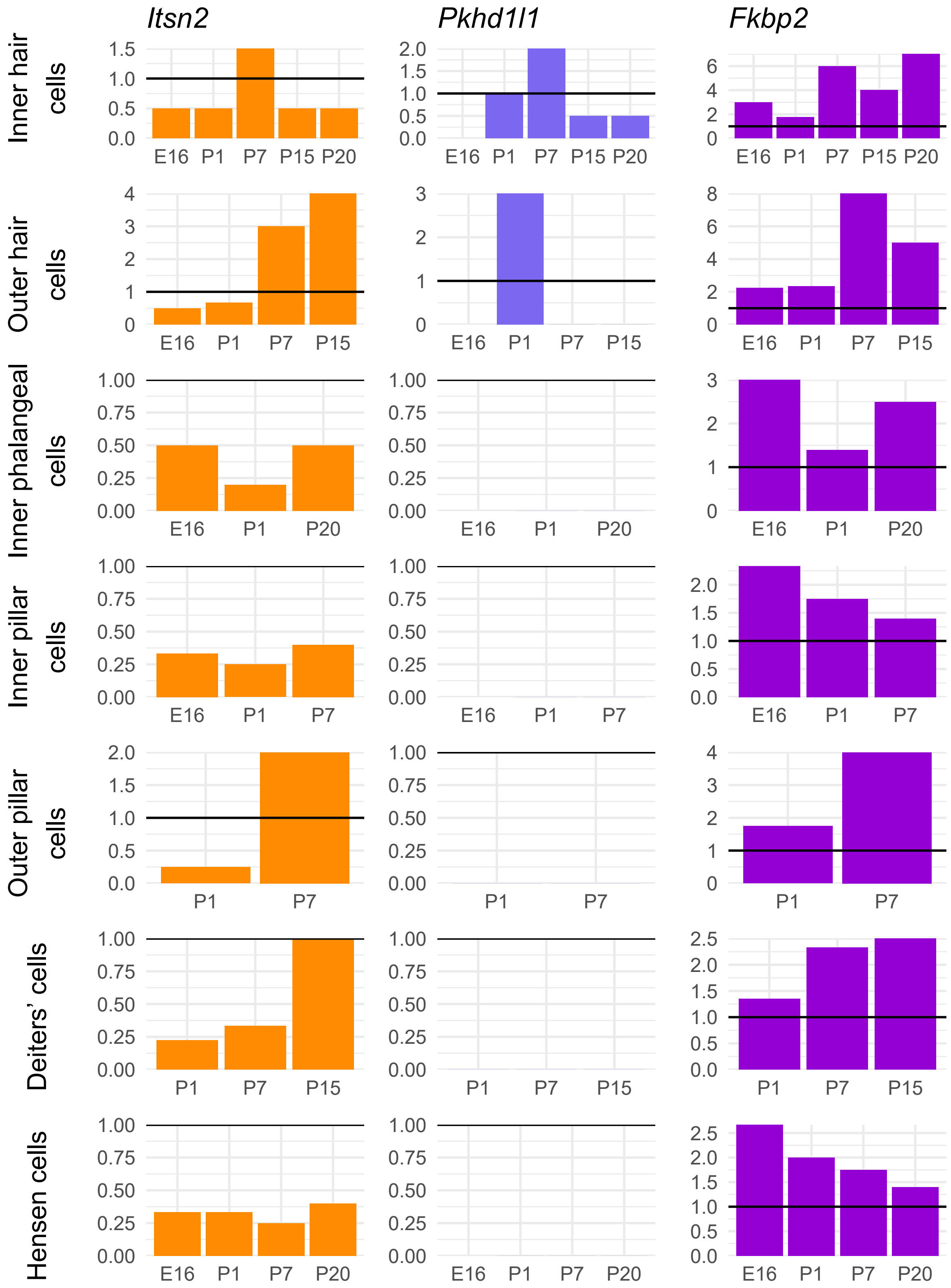

Itsn2
Pkhd1l1
Fkbp2
Inner hair cells
Outer hair cells
Inner phalangeal cells
Inner pillar cells
Outer pillar cells
Deiters' cells
Hensen cells
E16
P1
P7
P15
P20

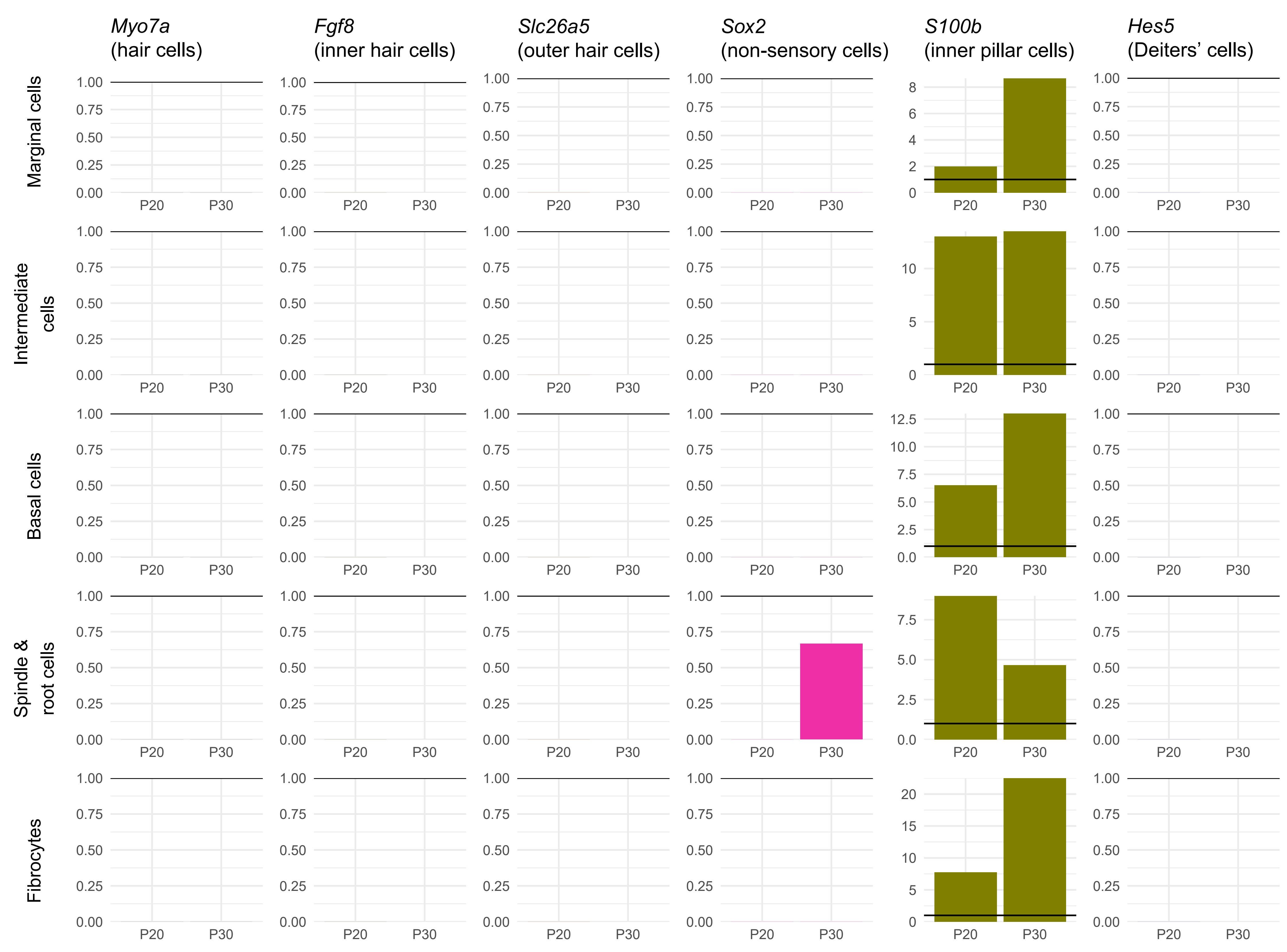
Myo7a (hair cells)
Fgf8 (inner hair cells)
Slc26a5 (outer hair cells)
Sox2 (non-sensory cells)
S100b (inner pillar cells)
Hes5 (Deiters' cells)
Marginal cells
Intermediate cells
Basal cells
Spindle & root cells
Fibrocytes
P20
P30

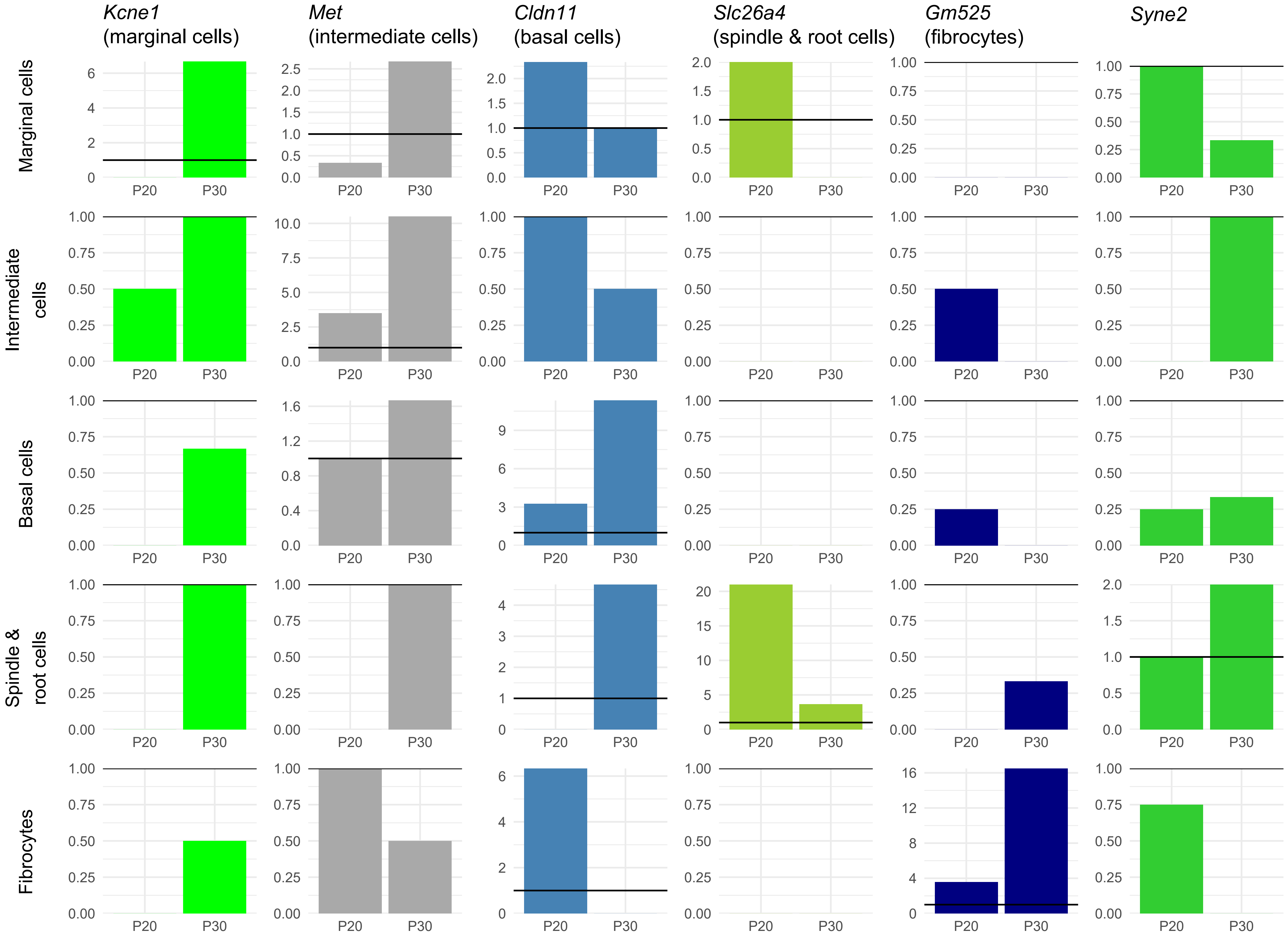

Kcne1
(marginal cells)
Met
(intermediate cells)
Cldn11
(basal cells)
Slc26a4
(spindle & root cells)
Gm525
(fibrocytes)
Syne2
Marginal cells
Intermediate cells
Basal cells
Spindle & root cells
Fibrocytes
P20
P30

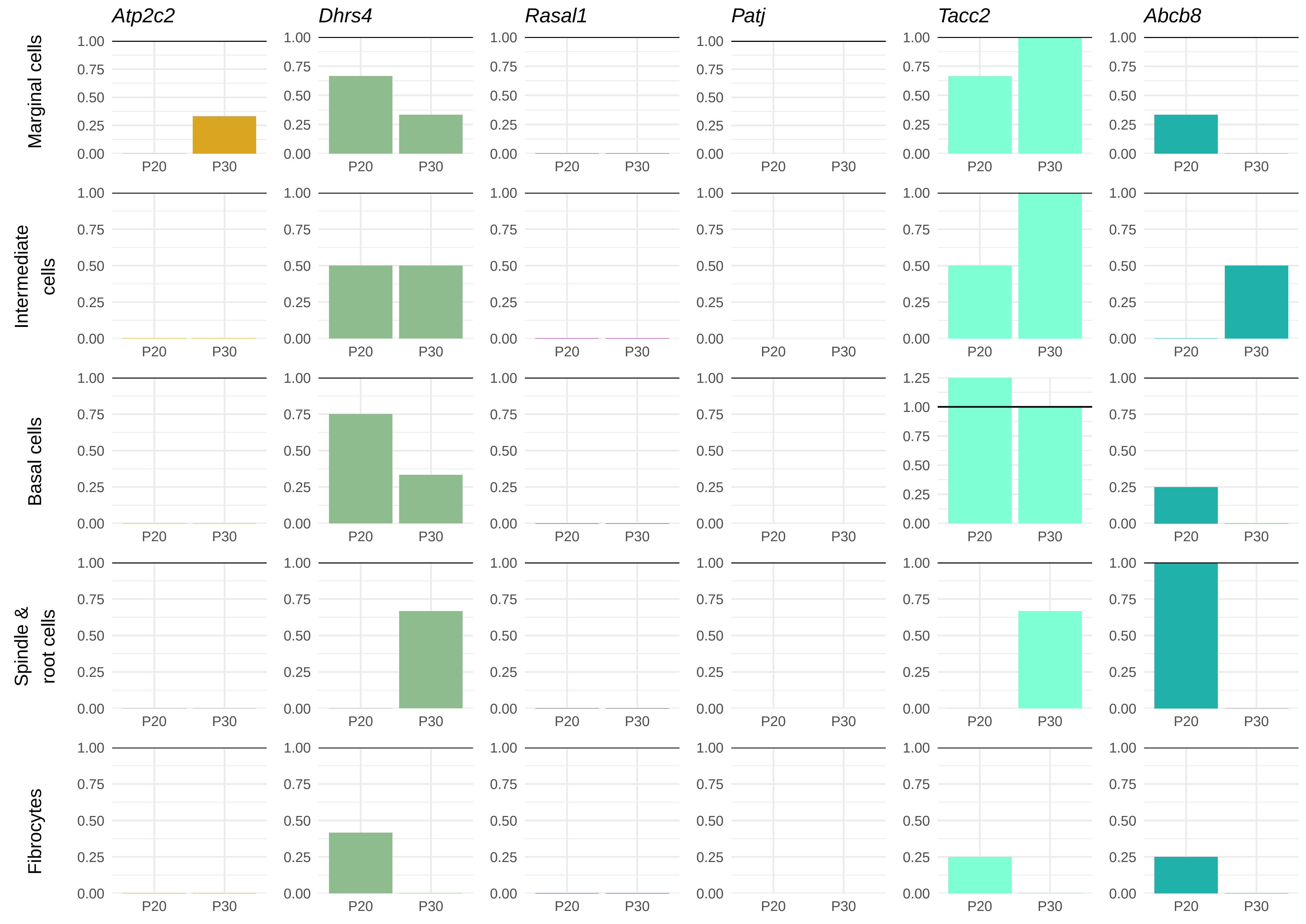

Atp2c2
Dhrs4
Rasal1
Patj
Tacc2
Abcb8
Marginal cells
Intermediate cells
Basal cells
Spindle & root cells
Fibrocytes
0.00
0.25
0.50
0.75
1.00
1.25
P20
P30

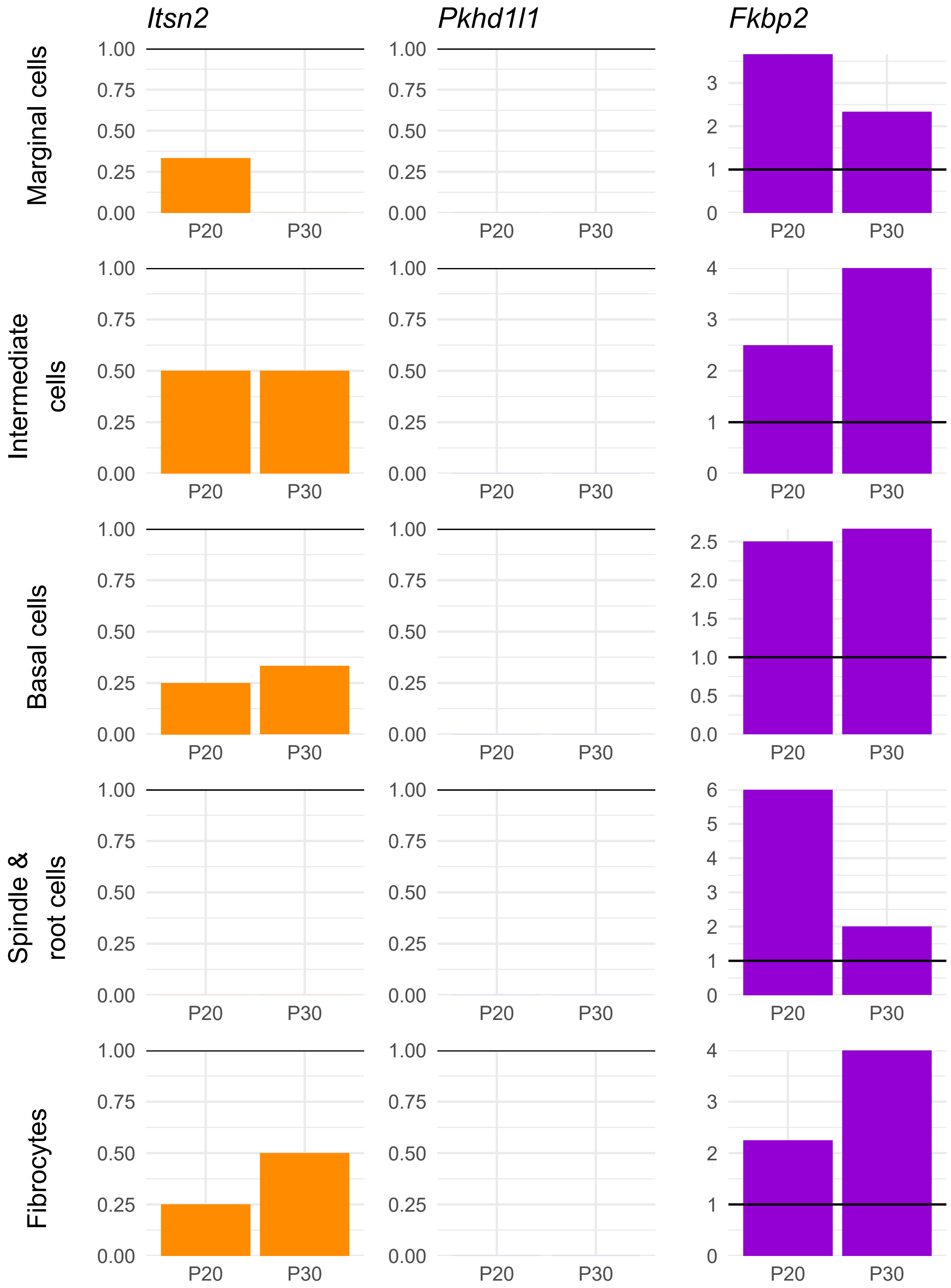

Itsn2
Pkhd1l1
Fkbp2
Marginal cells
Intermediate cells
Basal cells
Spindle & root cells
Fibrocytes
P20
P30

Suppl. Figure 5. Plots of gene expression in different cell types of the mouse inner ear, from single cell RNAseq data. These are genes identified in 3 cohorts (MUSC, TwinsUK and UK Biobank (Lewis et al. 2022)). Expression was normalised to *Hprt* (represented by a horizontal line at y=1 on each plot). Marker genes have been included for comparison (*Myo7a* (hair cells), *Fgf8* (inner hair cells), *Slc26a5* (outer hair cells), *Sox2* (non-sensory cells), *S100b* (inner pillar cells), *Hes5* (Deiters' cells), *Kcne1* (marginal cells), *Met* (intermediate cells), *Cldn11* (basal cells), *Slc26a4* (spindle and root cells), *Gm525* (fibrocytes)). Datasets were obtained from the gEAR (http://umgear.org) (Kolla et al. 2020; Korrapati et al. 2019; Ranum et al. 2019; Xue et al. 2021).

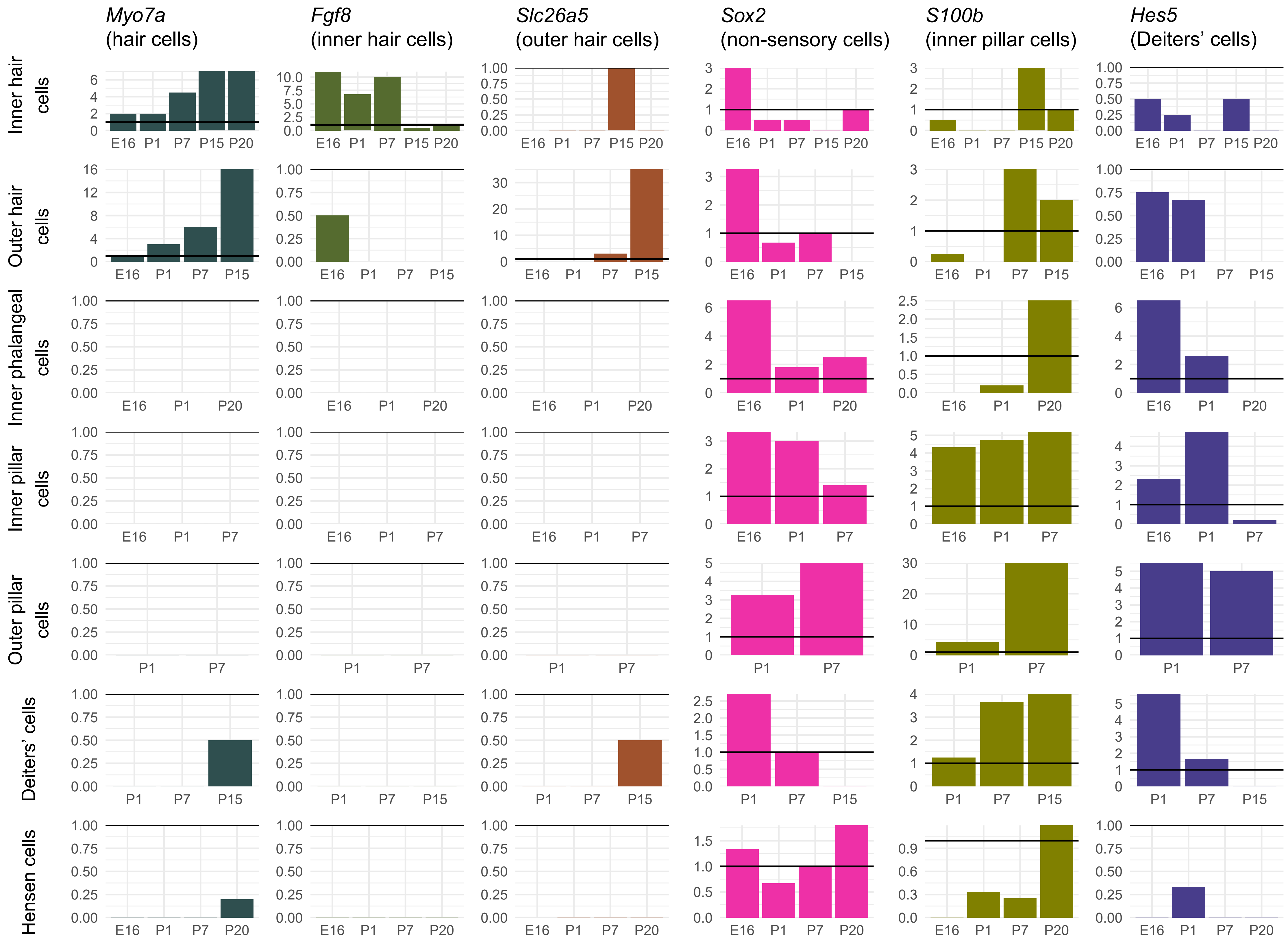

Myo7a (hair cells)
Fgf8 (inner hair cells)
Slc26a5 (outer hair cells)
Sox2 (non-sensory cells)
S100b (inner pillar cells)
Hes5 (Deiters' cells)
Inner hair cells
Outer hair cells
Inner phalangeal cells
Inner pillar cells
Outer pillar cells
Deiters' cells
Hensen cells
E16 P1 P7 P15 P20
E16 P1 P7 P15
E16 P1 P20
E16 P1 P7
P1 P7
P1 P7 P15
E16 P1 P7 P20

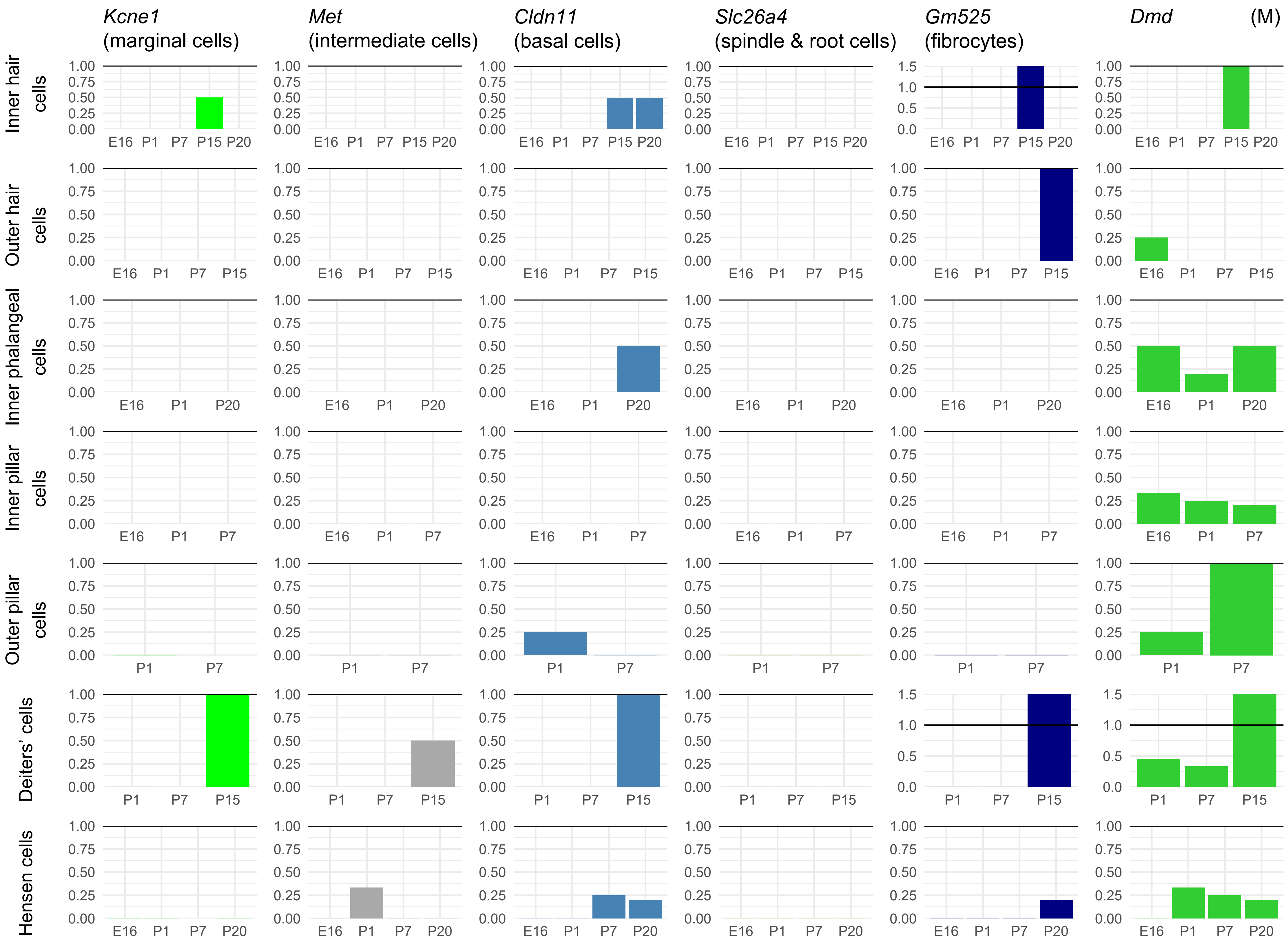

Kcne1
(marginal cells)
Met
(intermediate cells)
Cldn11
(basal cells)
Slc26a4
(spindle & root cells)
Gm525
(fibrocytes)
Dmd
(M)
Inner hair cells
Outer hair cells
Inner phalangeal cells
Inner pillar cells
Outer pillar cells
Deiters' cells
Hensen cells
E16
P1
P7
P15
P20

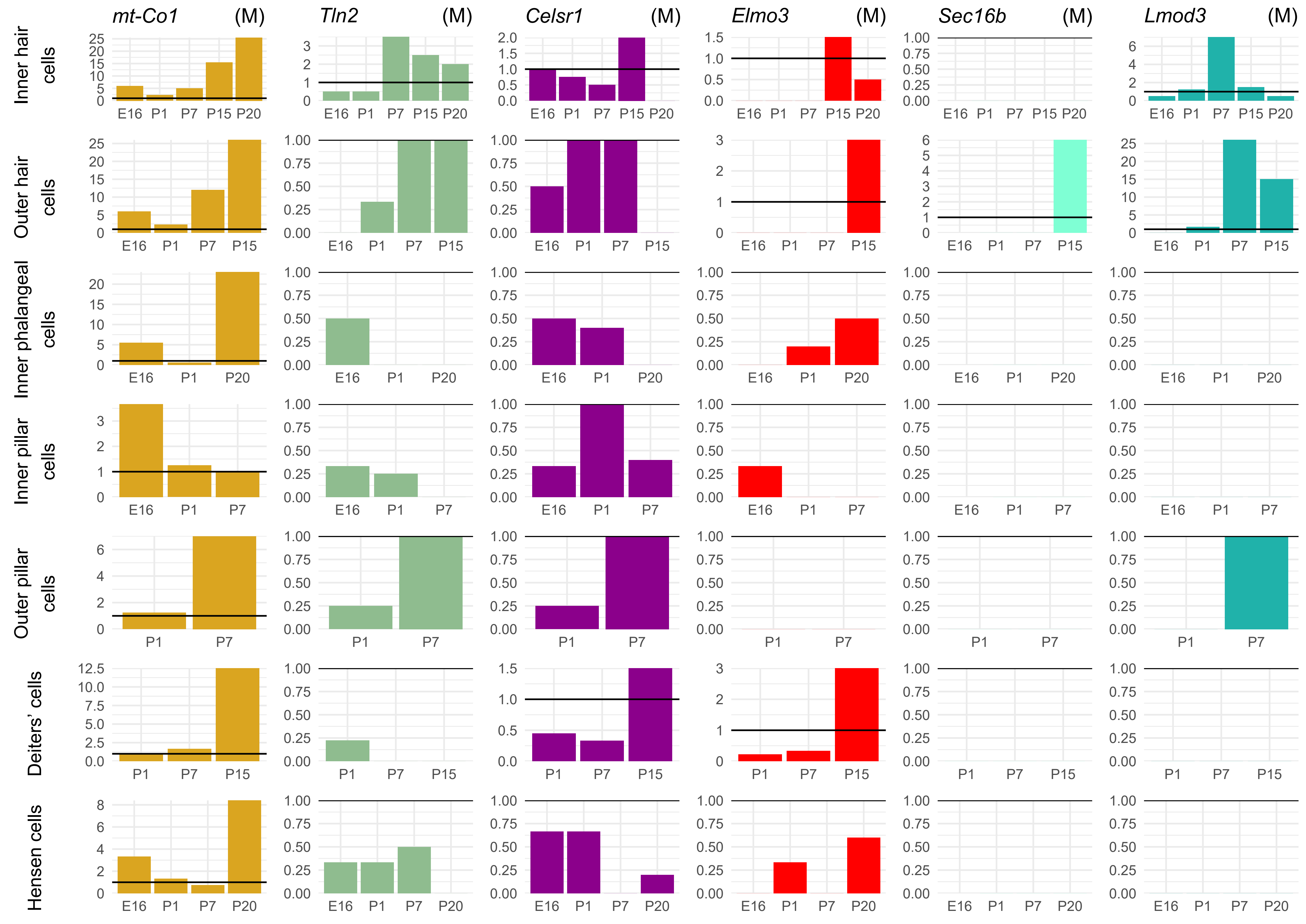

mt-Co1 (M)
Tln2 (M)
Celsr1 (M)
Elmo3 (M)
Sec16b (M)
Lmod3 (M)
Inner hair cells
Outer hair cells
Inner phalangeal cells
Inner pillar cells
Outer pillar cells
Deiters' cells
Hensen cells
E16
P1
P7
P15
P20

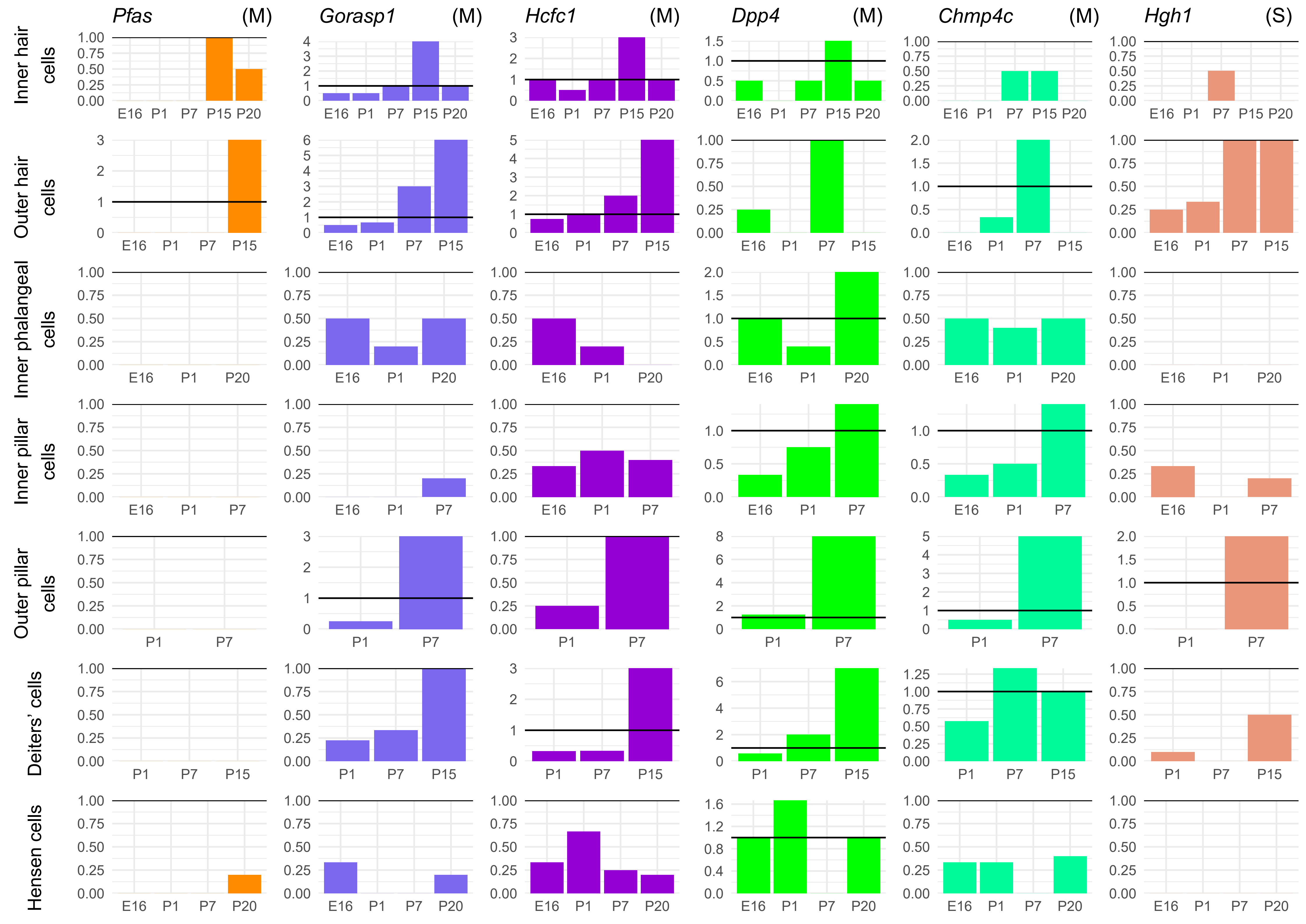
Pfas (M)
Gorasp1 (M)
Hcfc1 (M)
Dpp4 (M)
Chmp4c (M)
Hgh1 (S)
Inner hair cells
Outer hair cells
Inner phalangeal cells
Inner pillar cells
Outer pillar cells
Deiters' cells
Hensen cells
E16
P1
P7
P15
P20

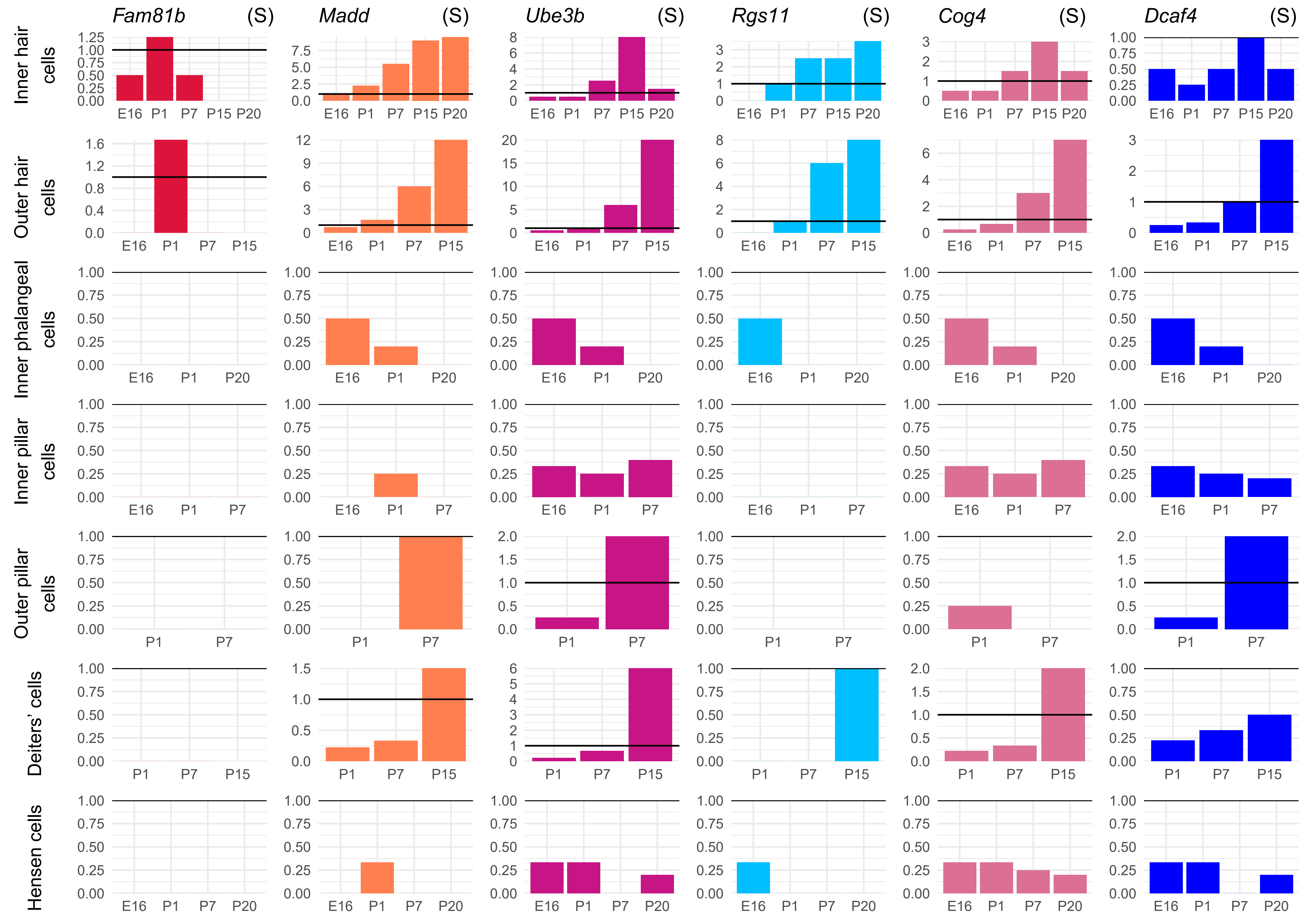

Fam81b (S)
Madd (S)
Ube3b (S)
Rgs11 (S)
Cog4 (S)
Dcaf4 (S)
Inner hair cells
Outer hair cells
Inner phalangeal cells
Inner pillar cells
Outer pillar cells
Deiters' cells
Hensen cells
E16 P1 P7 P15 P20

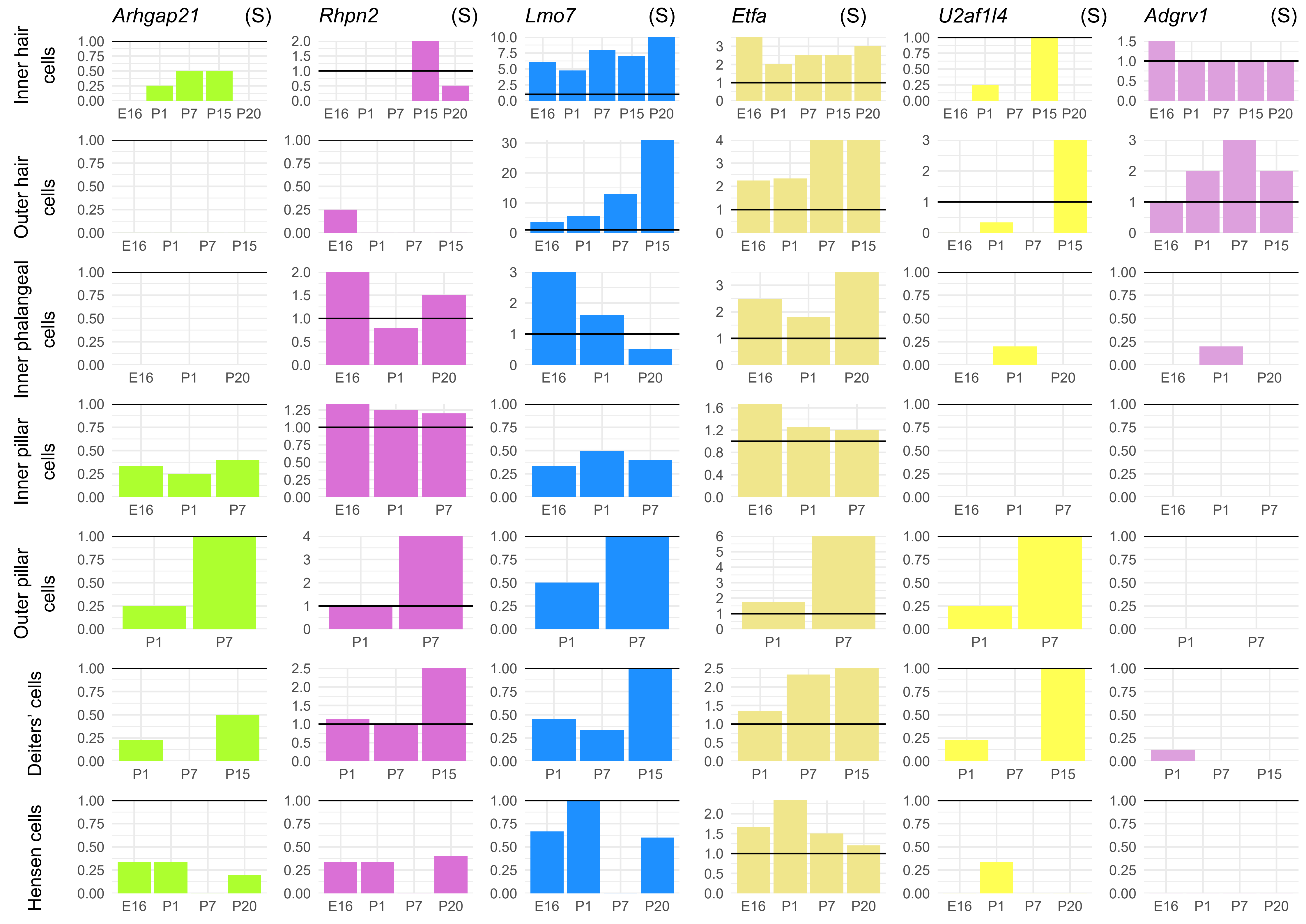

Arhgap21 (S)
Rhpn2 (S)
Lmo7 (S)
Etfa (S)
U2af1l4 (S)
Adgrv1 (S)
Inner hair cells
Outer hair cells
Inner phalangeal cells
Inner pillar cells
Outer pillar cells
Deiters' cells
Hensen cells
E16 P1 P7 P15 P20

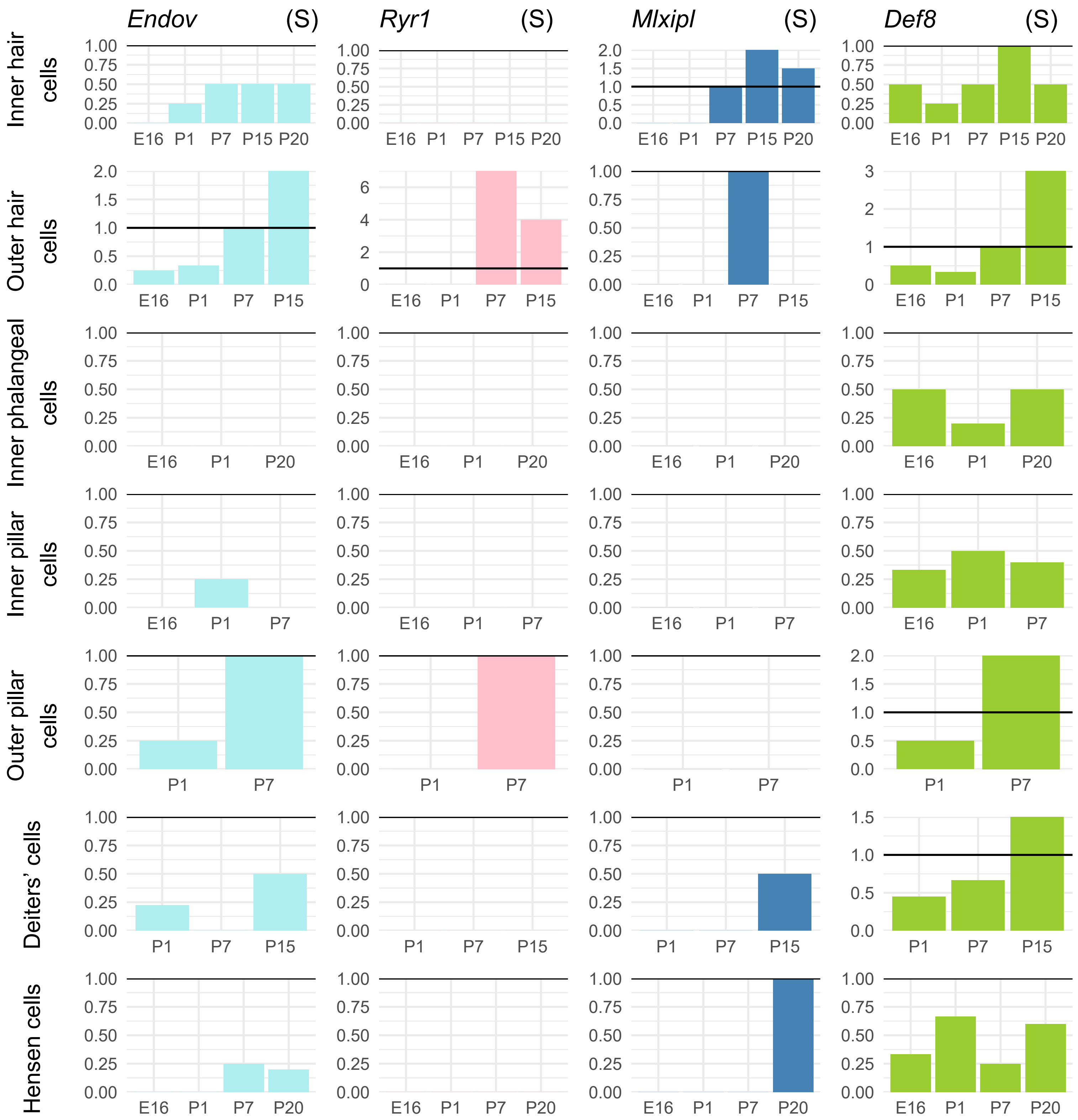
Endov (S)
Ryr1 (S)
Mlxipl (S)
Def8 (S)
Inner hair cells
Outer hair cells
Inner phalangeal cells
Inner pillar cells
Outer pillar cells
Deiters' cells
Hensen cells
E16
P1
P7
P15
P20
0.00
0.25
0.50
0.75
1.00
0.0
0.5
1.0
1.5
2.0
0
1
2
3
4
6

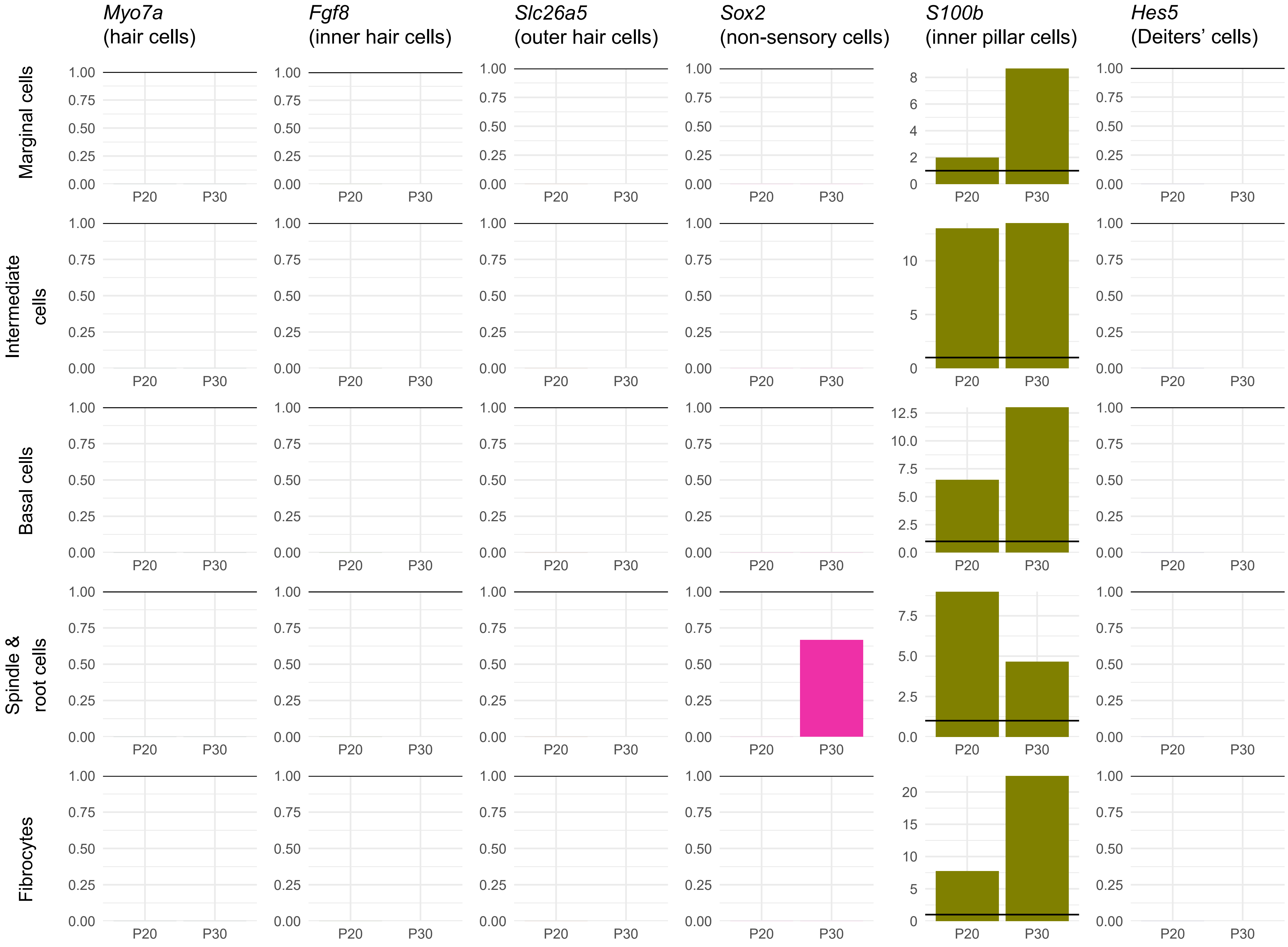

Myo7a
(hair cells)
Fgf8
(inner hair cells)
Slc26a5
(outer hair cells)
Sox2
(non-sensory cells)
S100b
(inner pillar cells)
Hes5
(Deiters' cells)
Marginal cells
Intermediate cells
Basal cells
Spindle & root cells
Fibrocytes
1.00
0.75
0.50
0.25
0.00
P20
P30

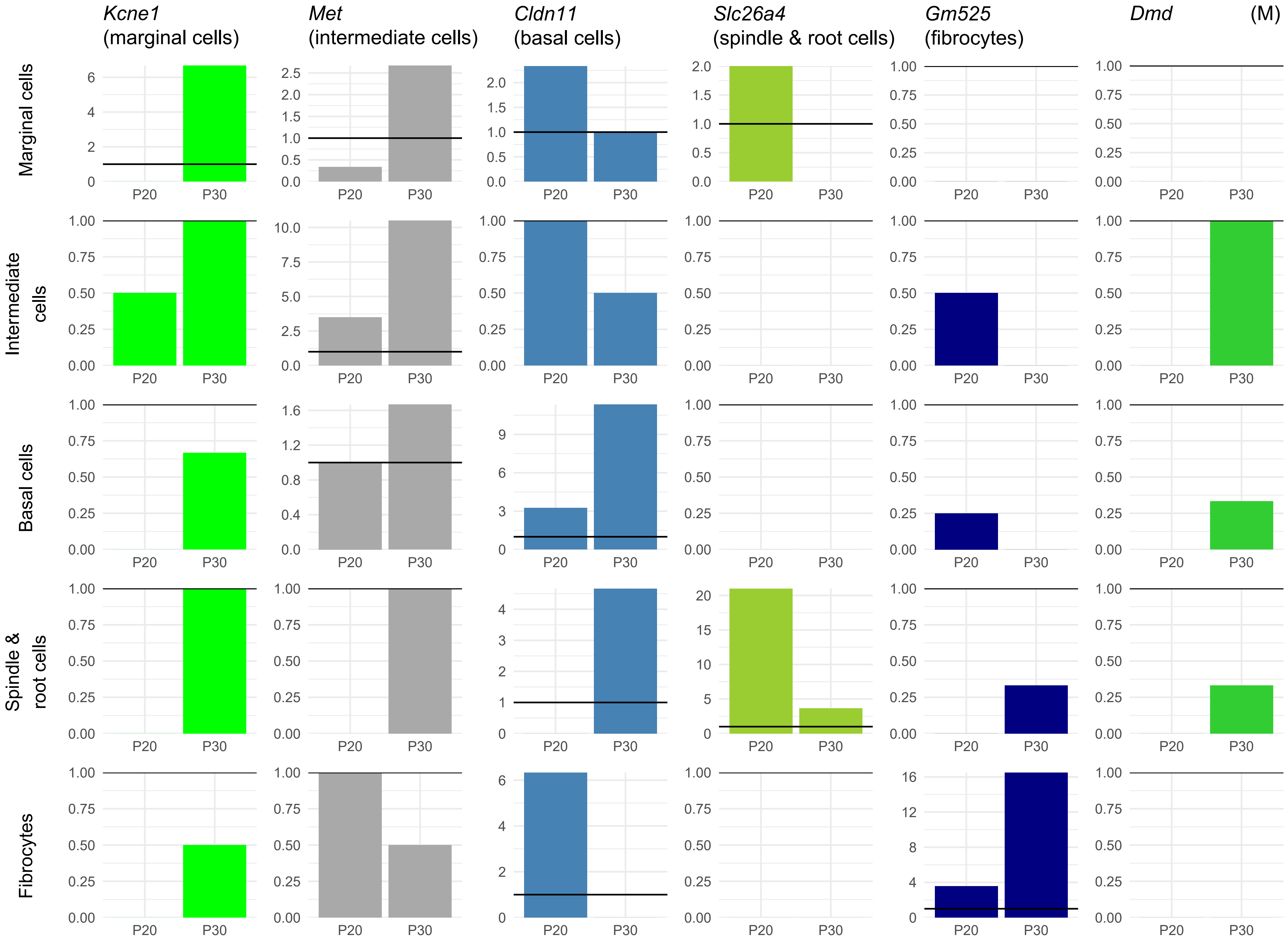
(M)
Kcne1
(marginal cells)
Met
(intermediate cells)
Cldn11
(basal cells)
Slc26a4
(spindle & root cells)
Gm525
(fibrocytes)
Dmd
Marginal cells
Intermediate cells
Basal cells
Spindle & root cells
Fibrocytes
P20
P30

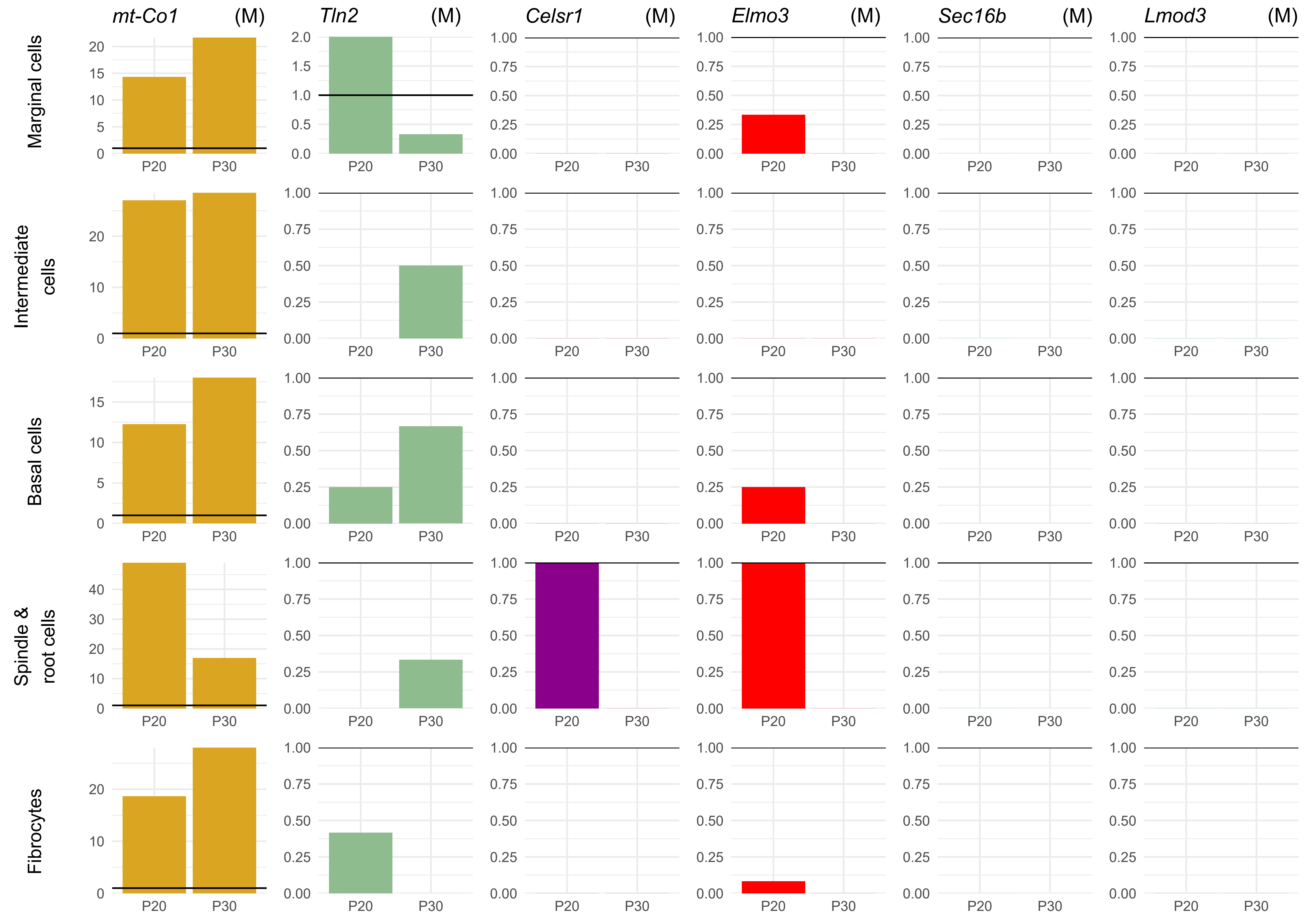

mt-Co1 (M)
Tln2 (M)
Celsr1 (M)
Elmo3 (M)
Sec16b (M)
Lmod3 (M)
Marginal cells
Intermediate cells
Basal cells
Spindle & root cells
Fibrocytes
P20
P30

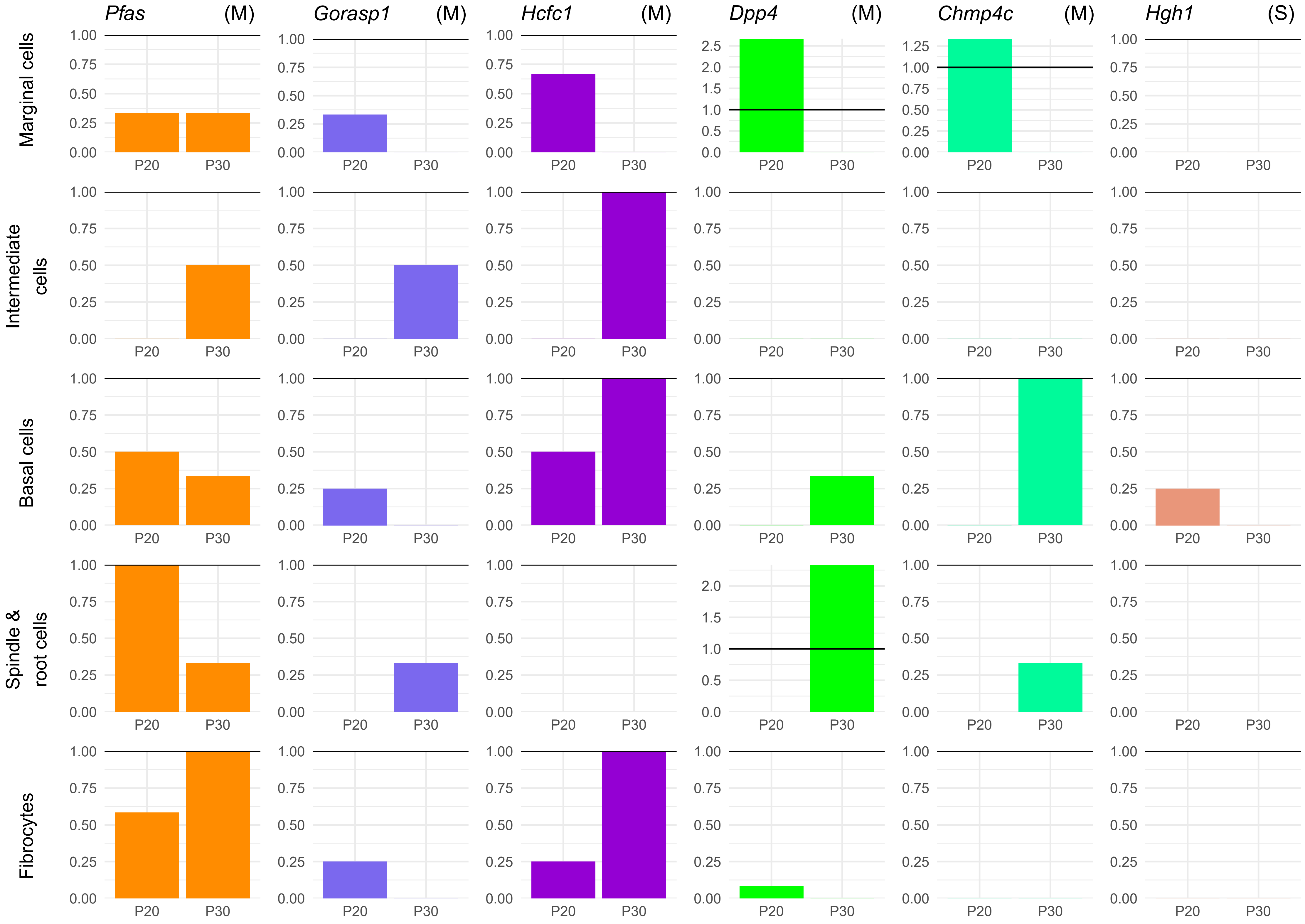

Pfas (M)
Gorasp1 (M)
Hcfc1 (M)
Dpp4 (M)
Chmp4c (M)
Hgh1 (S)
Marginal cells
Intermediate cells
Basal cells
Spindle & root cells
Fibrocytes
P20
P30

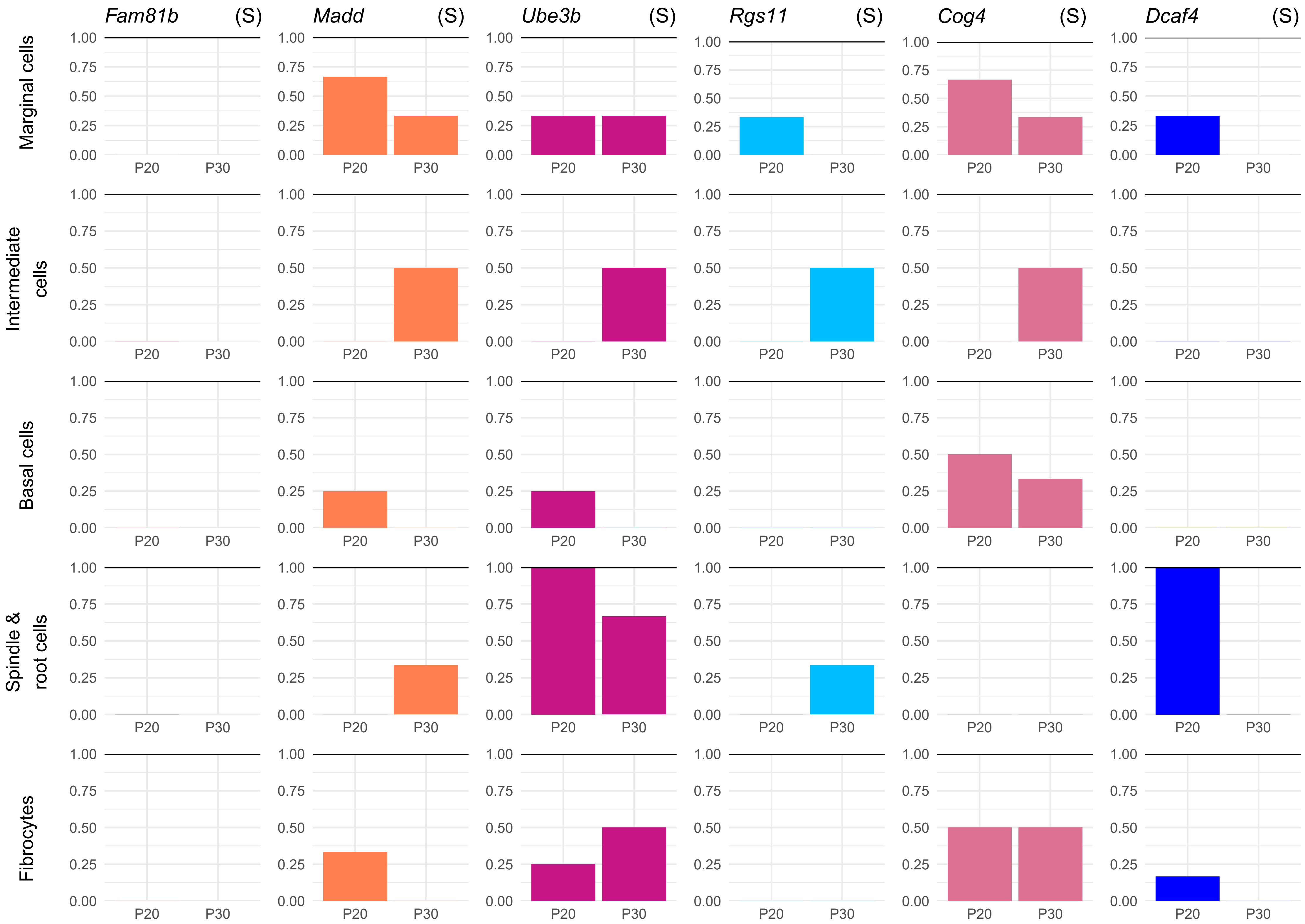

Fam81b (S)
Madd (S)
Ube3b (S)
Rgs11 (S)
Cog4 (S)
Dcaf4 (S)
Marginal cells
Intermediate cells
Basal cells
Spindle & root cells
Fibrocytes
1.00
0.75
0.50
0.25
0.00
P20
P30

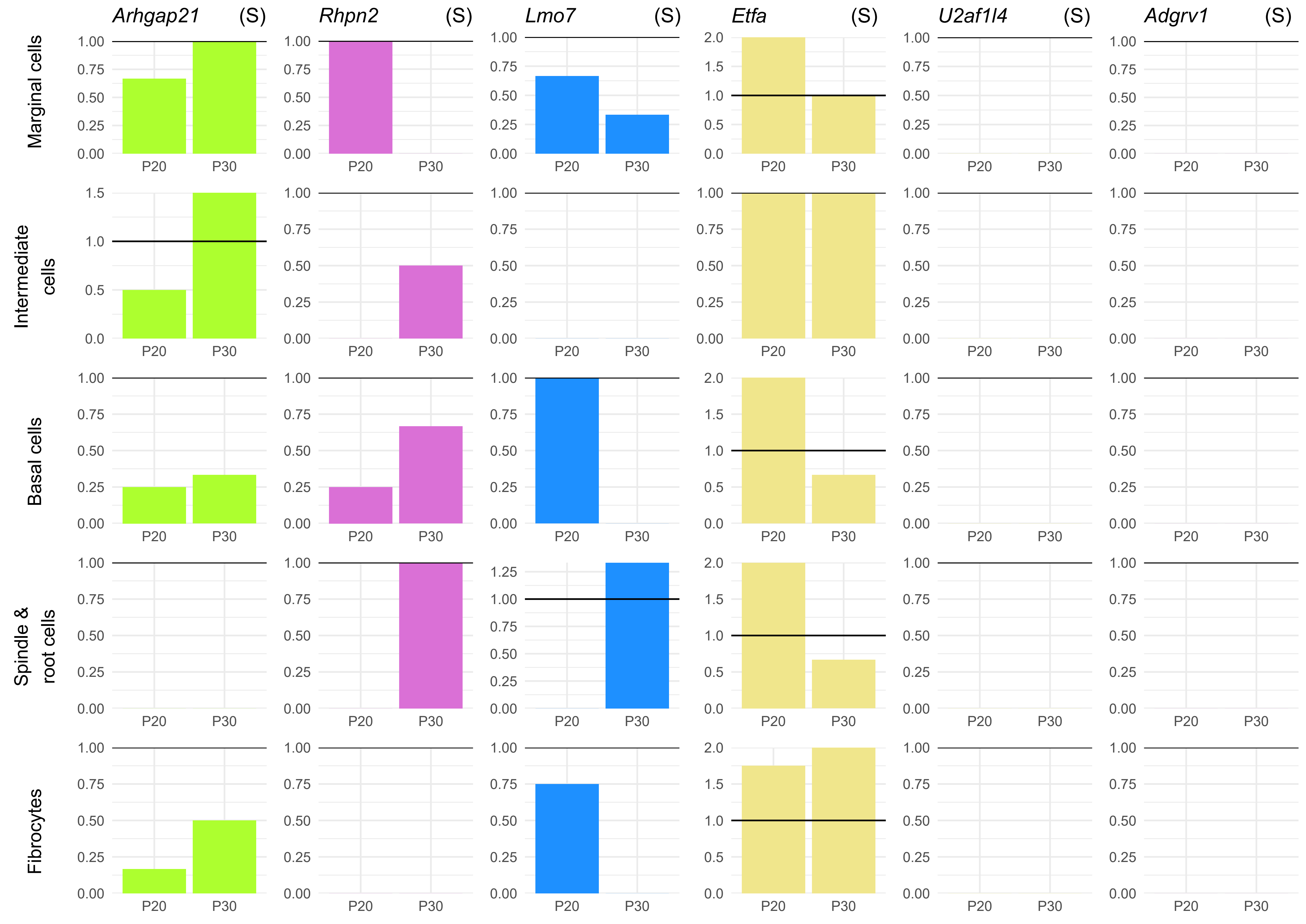
Arhgap21 (S)
Rhpn2 (S)
Lmo7 (S)
Etfa (S)
U2af1l4 (S)
Adgrv1 (S)
Marginal cells
Intermediate cells
Basal cells
Spindle & root cells
Fibrocytes
P20
P30

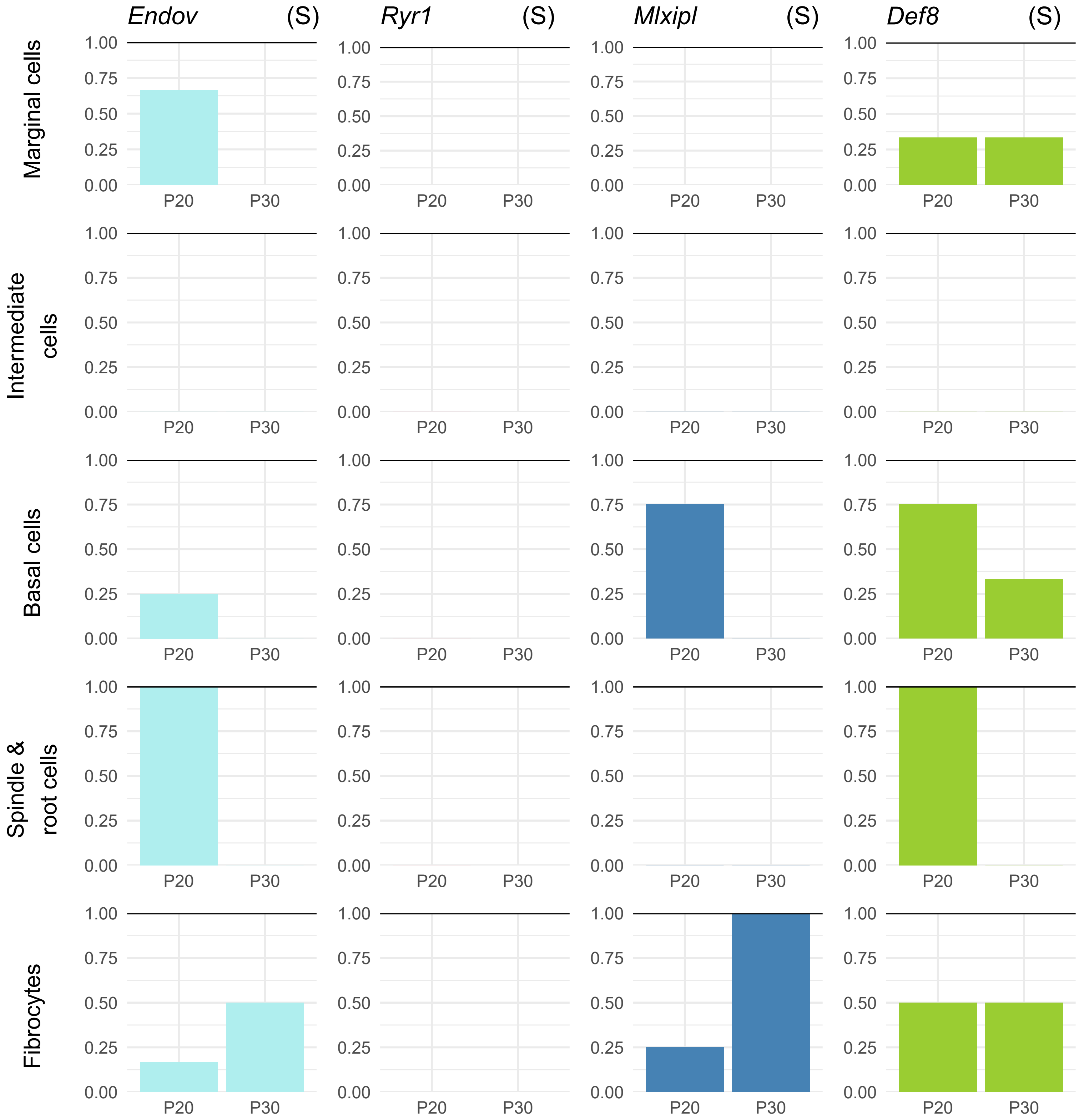

Endov
(S)
Ryr1
(S)
Mlxipl
(S)
Def8
(S)
Marginal cells
Intermediate cells
Basal cells
Spindle & root cells
Fibrocytes
1.00
0.75
0.50
0.25
0.00
P20
P30

Suppl. Figure 6. Plots of gene expression in different cell types of the mouse inner ear, from single cell RNAseq data. These are genes linked only to phenotypic subtypes in this study (M = Metabolic, S = Sensory). Expression was normalised to *Hprt* (represented by a horizontal line at y=1 on each plot). Marker genes have been included for comparison (*Myo7a* (hair cells), *Fgf8* (inner hair cells), *Slc26a5* (outer hair cells), *Sox2* (non-sensory cells), *S100b* (inner pillar cells), *Hes5* (Deiters' cells), *Kcne1* (marginal cells), *Met* (intermediate cells), *Cldn11* (basal cells), *Slc26a4* (spindle and root cells), *Gm525* (fibrocytes)). Datasets were obtained from the gEAR (http://umgear.org) (Kolla et al. 2020; Korrapati et al. 2019; Ranum et al. 2019; Xue et al. 2021).

MUSC cohort TwinsUK cohort

ATAD3B rs1622213 G>A

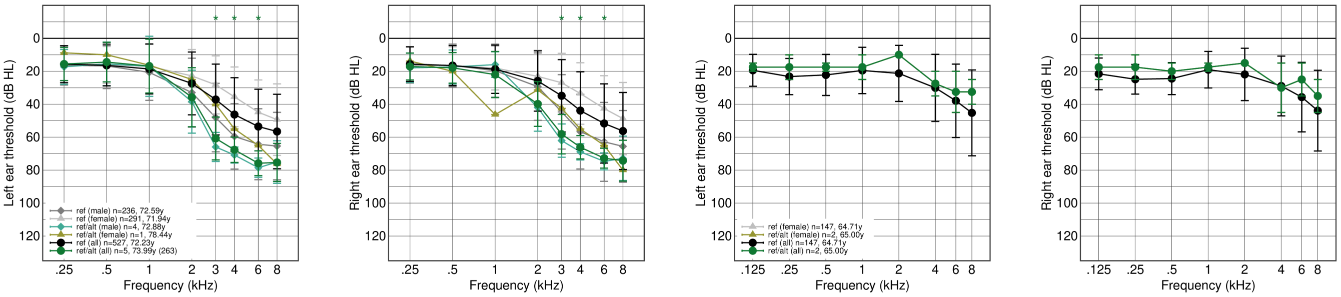

SYNC rs41265855 G>A

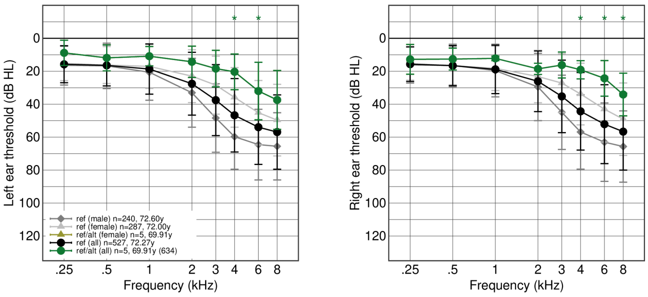

TCEANC2 rs41294786 C>G

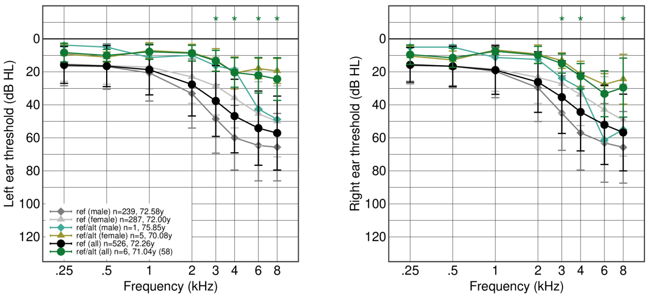

KIRREL1 rs139995772 C>T

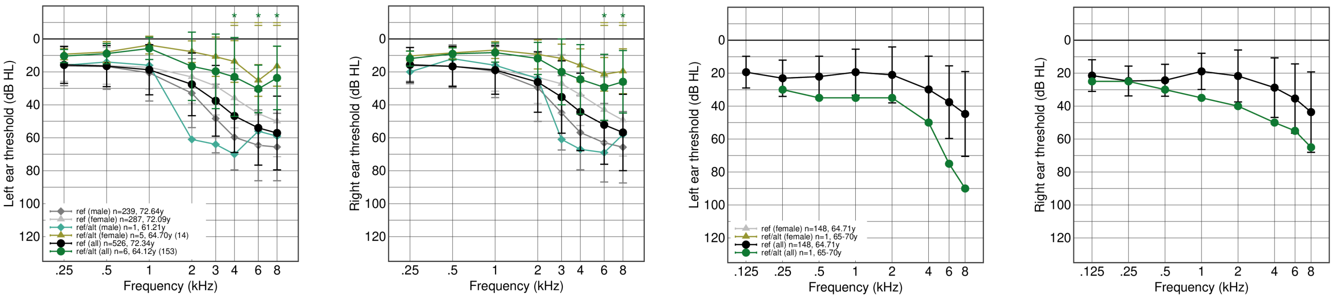

CAPN9 rs28359655 C>T

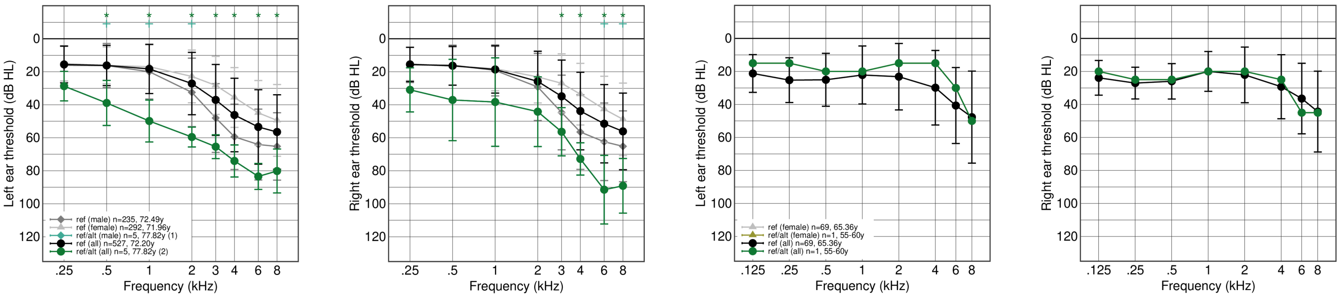

HS6ST1 . G>T

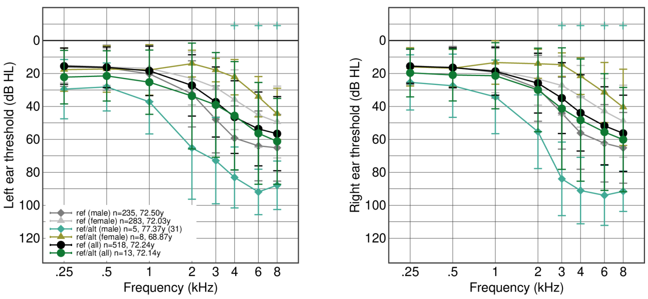

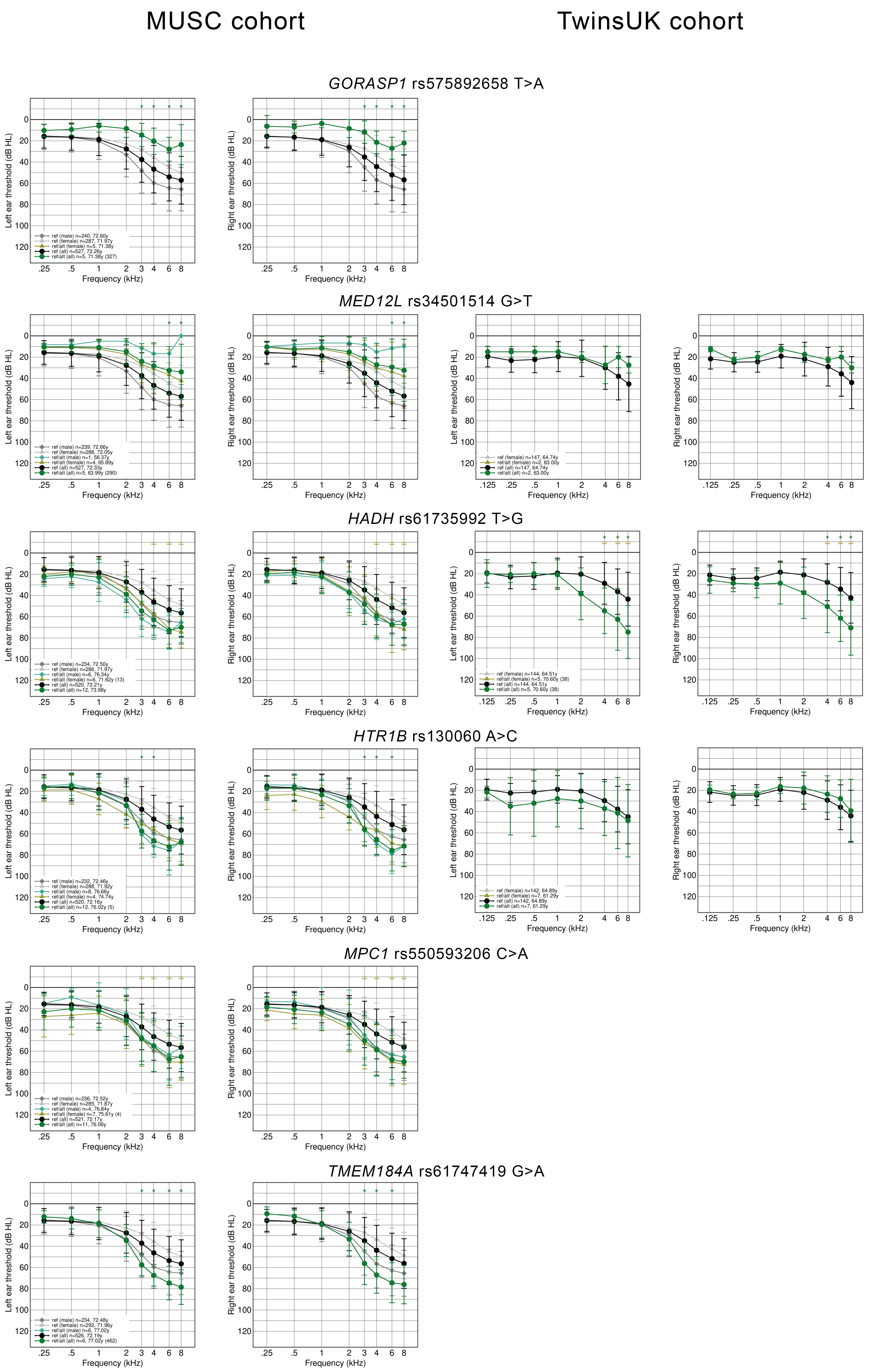
MUSC cohort
TwinsUK cohort
GORASP1 rs575892658 T>A
ref (male) n=240, 72.60y
ref (female) n=287, 71.97y
ref/alt (female) n=5, 71.38y
ref (all) n=527, 72.26y
ref/alt (all) n=5, 71.38y (327)
MED12L rs34501514 G>T
ref (male) n=239, 72.66y
ref (female) n=288, 72.05y
ref/alt (male) n=1, 56.37y
ref/alt (female) n=4, 65.89y
ref (all) n=527, 72.33y
ref/alt (all) n=5, 63.99y (290)
ref (female) n=147, 64.74y
ref/alt (female) n=2, 63.00y
ref (all) n=147, 64.74y
ref/alt (all) n=2, 63.00y
HADH rs61735992 T>G
ref (male) n=234, 72.50y
ref (female) n=286, 71.97y
ref/alt (male) n=6, 76.34y
ref/alt (female) n=6, 71.62y (13)
ref (all) n=520, 72.21y
ref/alt (all) n=12, 73.98y
ref (female) n=144, 64.51y
ref/alt (female) n=5, 70.60y (38)
ref (all) n=144, 64.51y
ref/alt (all) n=5, 70.60y (38)
HTR1B rs130060 A>C
ref (male) n=232, 72.46y
ref (female) n=288, 71.92y
ref/alt (male) n=8, 76.66y
ref/alt (female) n=4, 74.74y
ref (all) n=520, 72.16y
ref/alt (all) n=12, 76.02y (5)
ref (female) n=142, 64.89y
ref/alt (female) n=7, 61.29y
ref (all) n=142, 64.89y
ref/alt (all) n=7, 61.29y
MPC1 rs550593206 C>A
ref (male) n=236, 72.52y
ref (female) n=285, 71.87y
ref/alt (male) n=4, 76.84y
ref/alt (female) n=7, 75.61y (4)
ref (all) n=521, 72.17y
ref/alt (all) n=11, 76.06y
TMEM184A rs61747419 G>A
ref (male) n=234, 72.48y
ref (female) n=292, 71.96y
ref/alt (male) n=6, 77.02y
ref (all) n=526, 72.19y
ref/alt (all) n=6, 77.02y (462)
Left ear threshold (dB HL)
Right ear threshold (dB HL)
Frequency (kHz)

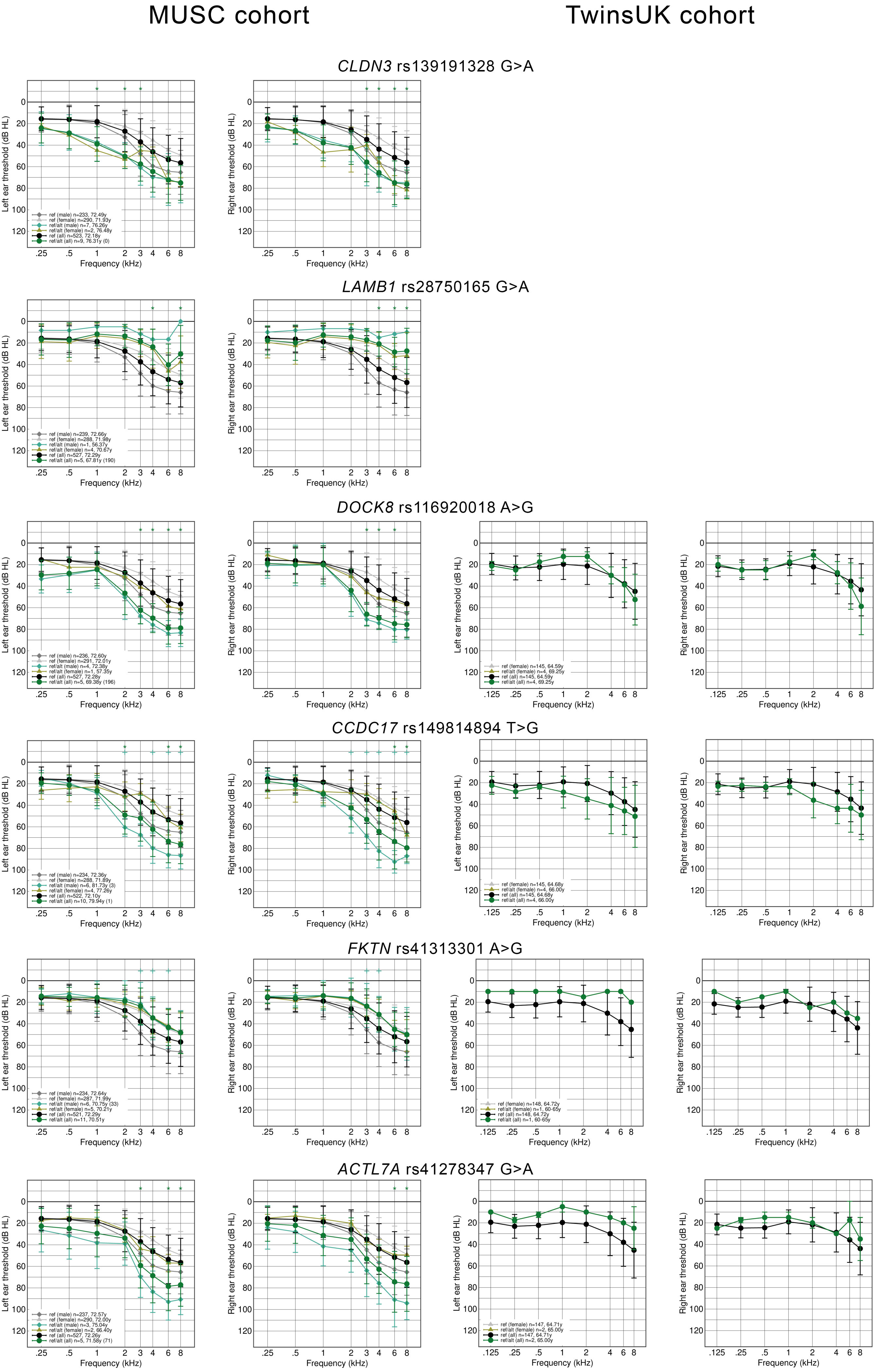
MUSC cohort
TwinsUK cohort
CLDN3 rs139191328 G>A
LAMB1 rs28750165 G>A
DOCK8 rs116920018 A>G
CCDC17 rs149814894 T>G
FKTN rs41313301 A>G
ACTL7A rs41278347 G>A
Left ear threshold (dB HL)
Right ear threshold (dB HL)
Frequency (kHz)

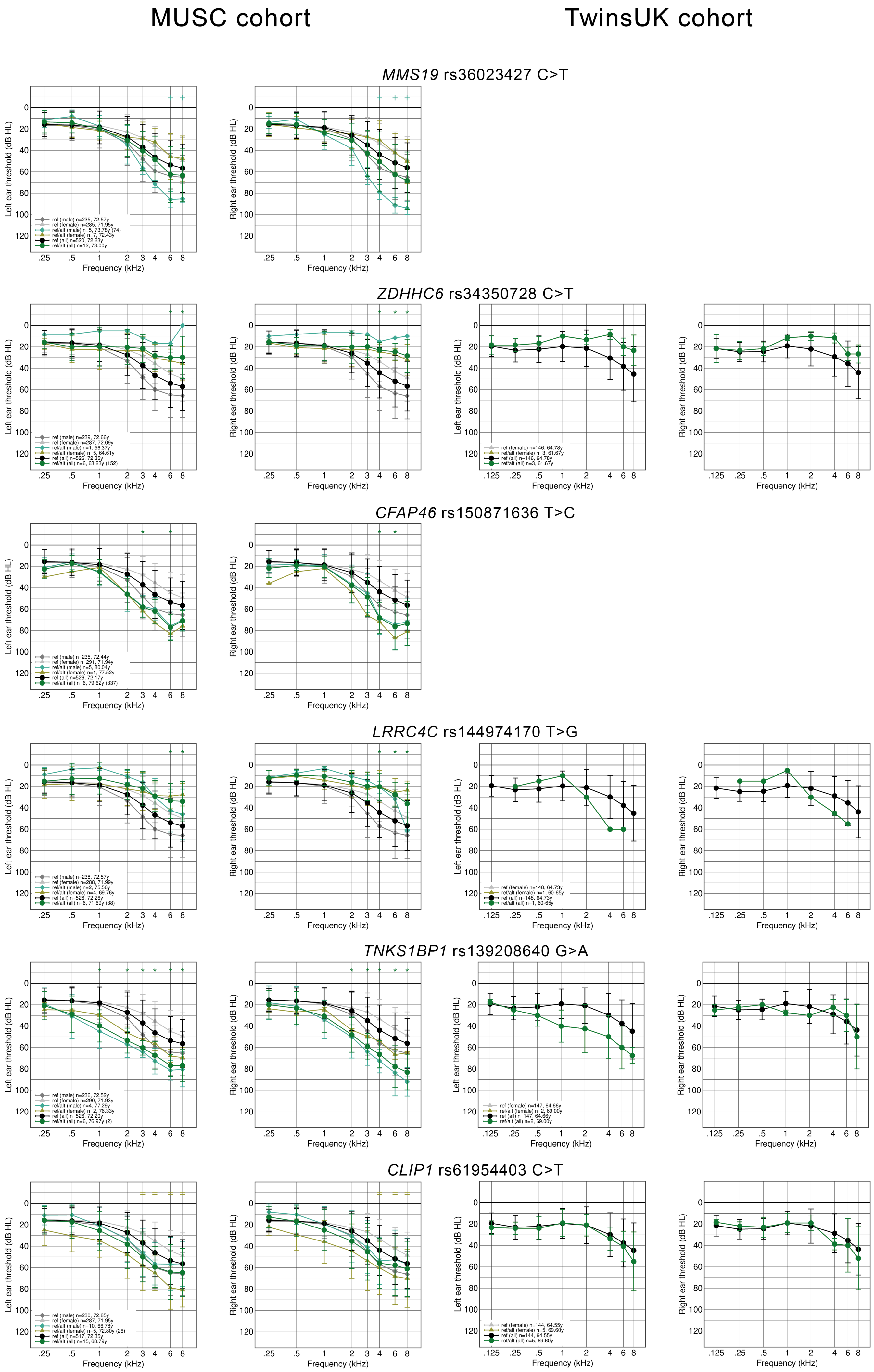

MUSC cohort
TwinsUK cohort
MMS19 rs36023427 C>T
ZDHHC6 rs34350728 C>T
CFAP46 rs150871636 T>C
LRRC4C rs144974170 T>G
TNKS1BP1 rs139208640 G>A
CLIP1 rs61954403 C>T
Left ear threshold (dB HL)
Right ear threshold (dB HL)
Frequency (kHz)
ref (male) n=235, 72.57y
ref (female) n=285, 71.95y
ref/alt (male) n=5, 73.78y (74)
ref/alt (female) n=7, 72.43y
ref (all) n=520, 72.23y
ref/alt (all) n=12, 73.00y
ref (male) n=239, 72.66y
ref (female) n=287, 72.09y
ref/alt (male) n=1, 56.37y
ref/alt (female) n=5, 64.61y
ref (all) n=526, 72.35y
ref/alt (all) n=6, 63.23y (152)
ref (female) n=146, 64.78y
ref/alt (female) n=3, 61.67y
ref (all) n=146, 64.78y
ref/alt (all) n=3, 61.67y
ref (male) n=235, 72.44y
ref (female) n=291, 71.94y
ref/alt (male) n=5, 80.04y
ref/alt (female) n=1, 77.52y
ref (all) n=526, 72.17y
ref/alt (all) n=6, 79.62y (337)
ref (male) n=238, 72.57y
ref (female) n=288, 71.99y
ref/alt (male) n=2, 75.56y
ref/alt (female) n=4, 69.76y
ref (all) n=526, 72.26y
ref/alt (all) n=6, 71.69y (38)
ref (female) n=148, 64.73y
ref/alt (female) n=1, 60-65y
ref (all) n=148, 64.73y
ref/alt (all) n=1, 60-65y
ref (male) n=236, 72.52y
ref (female) n=290, 71.93y
ref/alt (male) n=4, 77.29y
ref/alt (female) n=2, 76.33y
ref (all) n=526, 72.20y
ref/alt (all) n=6, 76.97y (2)
ref (female) n=147, 64.66y
ref/alt (female) n=2, 69.00y
ref (all) n=147, 64.66y
ref/alt (all) n=2, 69.00y
ref (male) n=230, 72.85y
ref (female) n=287, 71.95y
ref/alt (male) n=10, 66.78y
ref/alt (female) n=5, 72.80y (26)
ref (all) n=517, 72.35y
ref/alt (all) n=15, 68.79y
ref (female) n=144, 64.55y
ref/alt (female) n=5, 69.60y
ref (all) n=144, 64.55y
ref/alt (all) n=5, 69.60y

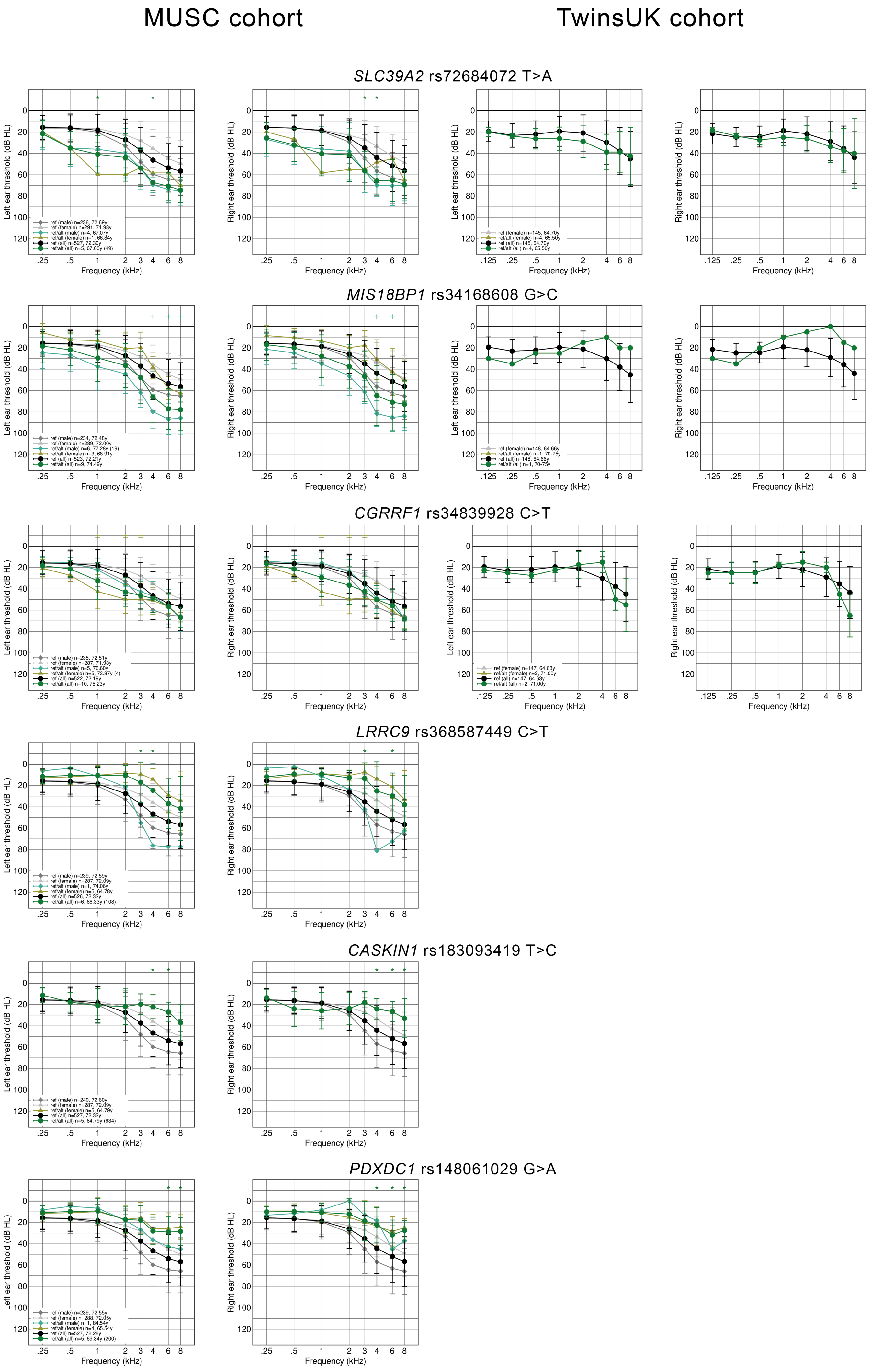
MUSC cohort
TwinsUK cohort
SLC39A2 rs72684072 T>A
ref (male) n=236, 72.69y
ref (female) n=291, 71.98y
ref/alt (male) n=4, 67.07y
ref/alt (female) n=1, 66.84y
ref (all) n=527, 72.30y
ref/alt (all) n=5, 67.03y (49)
ref (female) n=145, 64.70y
ref/alt (female) n=4, 65.50y
ref (all) n=145, 64.70y
ref/alt (all) n=4, 65.50y
MIS18BP1 rs34168608 G>C
ref (male) n=234, 72.48y
ref (female) n=289, 72.00y
ref/alt (male) n=6, 77.28y (19)
ref/alt (female) n=3, 68.91y
ref (all) n=523, 72.21y
ref/alt (all) n=9, 74.49y
ref (female) n=148, 64.66y
ref/alt (female) n=1, 70-75y
ref (all) n=148, 64.66y
ref/alt (all) n=1, 70-75y
CGRRF1 rs34839928 C>T
ref (male) n=235, 72.51y
ref (female) n=287, 71.93y
ref/alt (male) n=5, 76.60y
ref/alt (female) n=5, 73.87y (4)
ref (all) n=522, 72.19y
ref/alt (all) n=10, 75.23y
ref (female) n=147, 64.63y
ref/alt (female) n=2, 71.00y
ref (all) n=147, 64.63y
ref/alt (all) n=2, 71.00y
LRRC9 rs368587449 C>T
ref (male) n=239, 72.59y
ref (female) n=287, 72.09y
ref/alt (male) n=1, 74.06y
ref/alt (female) n=5, 64.78y
ref (all) n=526, 72.32y
ref/alt (all) n=6, 66.33y (108)
CASKIN1 rs183093419 T>C
ref (male) n=240, 72.60y
ref (female) n=287, 72.09y
ref/alt (female) n=5, 64.79y
ref (all) n=527, 72.32y
ref/alt (all) n=5, 64.79y (634)
PDXDC1 rs148061029 G>A
ref (male) n=239, 72.55y
ref (female) n=288, 72.05y
ref/alt (male) n=1, 84.54y
ref/alt (female) n=4, 65.54y
ref (all) n=527, 72.28y
ref/alt (all) n=5, 69.34y (200)
Left ear threshold (dB HL)
Right ear threshold (dB HL)
Frequency (kHz)

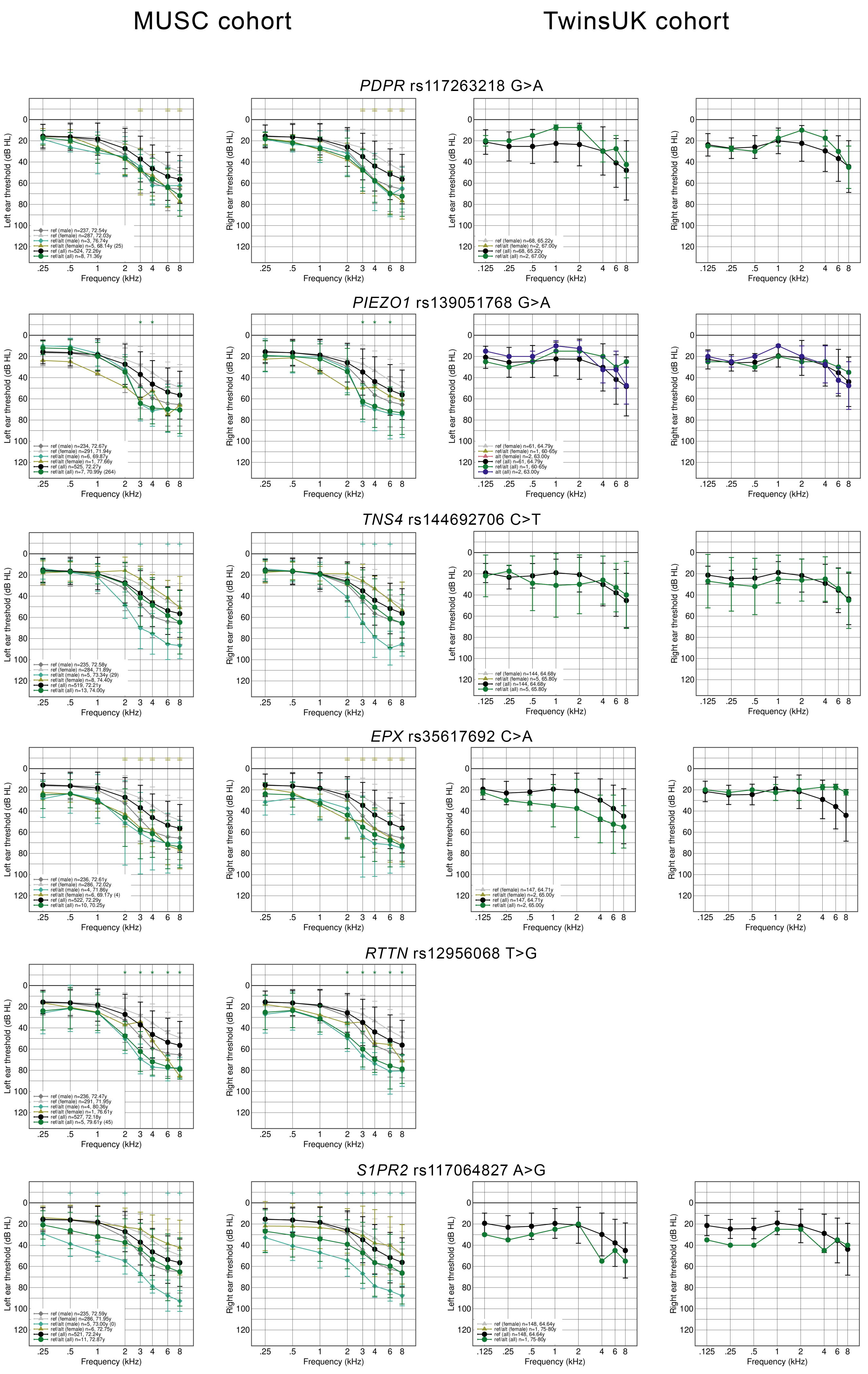
MUSC cohort
TwinsUK cohort
PDPR rs117263218 G>A
ref (male) n=237, 72.54y
ref (female) n=287, 72.03y
ref/alt (male) n=3, 76.74y
ref/alt (female) n=5, 68.14y (25)
ref (all) n=524, 72.26y
ref/alt (all) n=8, 71.36y
ref (female) n=68, 65.22y
ref/alt (female) n=2, 67.00y
ref (all) n=68, 65.22y
ref/alt (all) n=2, 67.00y
PIEZO1 rs139051768 G>A
ref (male) n=234, 72.67y
ref (female) n=291, 71.94y
ref/alt (male) n=6, 69.87y
ref/alt (female) n=1, 77.66y
ref (all) n=525, 72.27y
ref/alt (all) n=7, 70.99y (264)
ref (female) n=61, 64.79y
ref/alt (female) n=1, 60-65y
alt (female) n=2, 63.00y
ref (all) n=61, 64.79y
ref/alt (all) n=1, 60-65y
alt (all) n=2, 63.00y
TNS4 rs144692706 C>T
ref (male) n=235, 72.58y
ref (female) n=284, 71.89y
ref/alt (male) n=5, 73.34y (29)
ref/alt (female) n=8, 74.40y
ref (all) n=519, 72.21y
ref/alt (all) n=13, 74.00y
ref (female) n=144, 64.68y
ref/alt (female) n=5, 65.80y
ref (all) n=144, 64.68y
ref/alt (all) n=5, 65.80y
EPX rs35617692 C>A
ref (male) n=236, 72.61y
ref (female) n=286, 72.02y
ref/alt (male) n=4, 71.86y
ref/alt (female) n=6, 69.17y (4)
ref (all) n=522, 72.29y
ref/alt (all) n=10, 70.25y
ref (female) n=147, 64.71y
ref/alt (female) n=2, 65.00y
ref (all) n=147, 64.71y
ref/alt (all) n=2, 65.00y
RTTN rs12956068 T>G
ref (male) n=236, 72.47y
ref (female) n=291, 71.95y
ref/alt (male) n=4, 80.36y
ref/alt (female) n=1, 76.61y
ref (all) n=527, 72.18y
ref/alt (all) n=5, 79.61y (45)
S1PR2 rs117064827 A>G
ref (male) n=235, 72.59y
ref (female) n=286, 71.95y
ref/alt (male) n=5, 73.00y (0)
ref/alt (female) n=6, 72.75y
ref (all) n=521, 72.24y
ref/alt (all) n=11, 72.87y
ref (female) n=148, 64.64y
ref/alt (female) n=1, 75-80y
ref (all) n=148, 64.64y
ref/alt (all) n=1, 75-80y
Left ear threshold (dB HL)
Right ear threshold (dB HL)
Frequency (kHz)

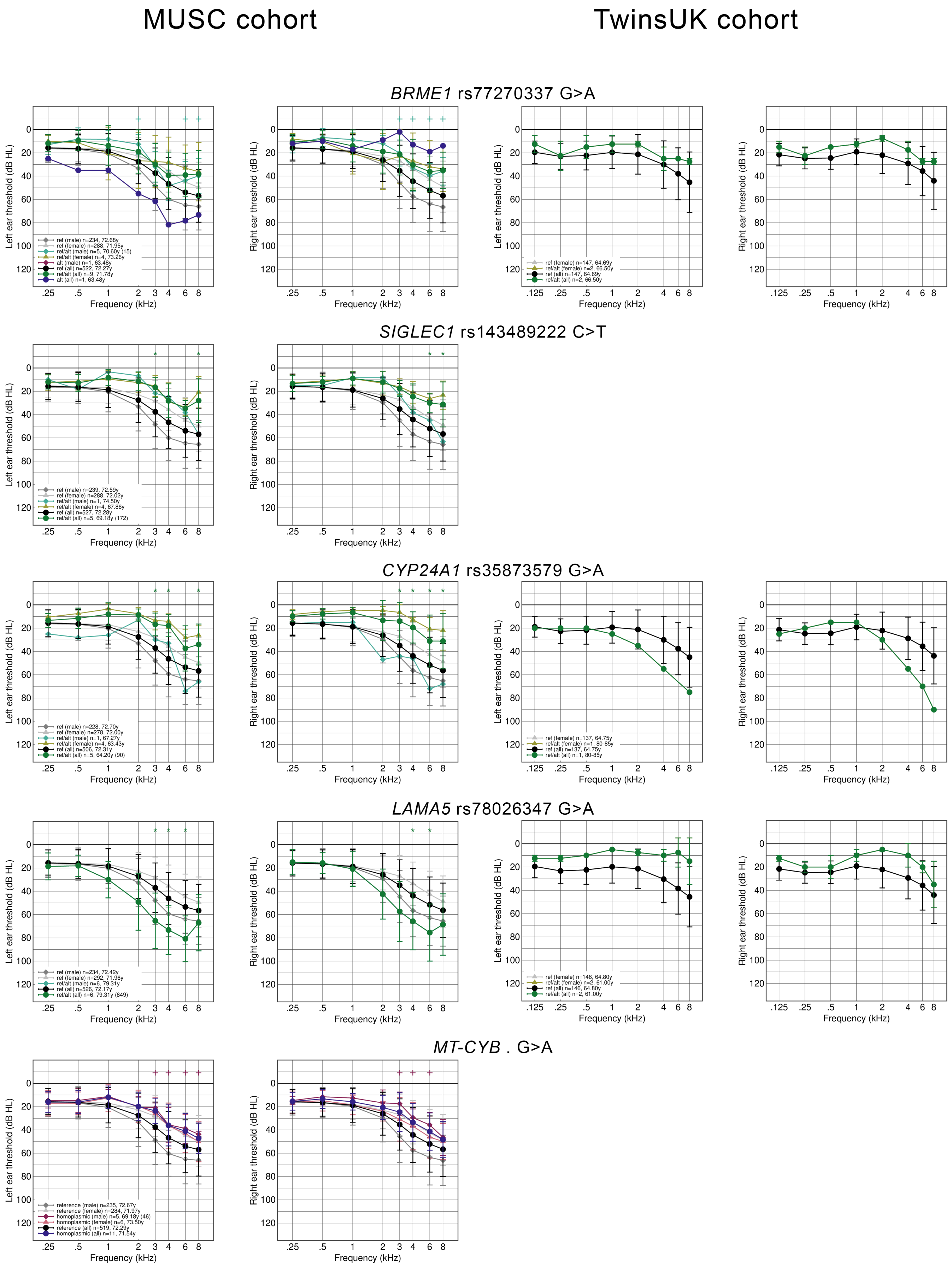
MUSC cohort
TwinsUK cohort
BRME1 rs77270337 G>A
SIGLEC1 rs143489222 C>T
CYP24A1 rs35873579 G>A
LAMA5 rs78026347 G>A
MT-CYB . G>A
Left ear threshold (dB HL)
Right ear threshold (dB HL)
Frequency (kHz)
ref (male) n=234, 72.68y
ref (female) n=288, 71.95y
ref/alt (male) n=5, 70.60y (15)
ref/alt (female) n=4, 73.26y
alt (male) n=1, 63.48y
ref (all) n=522, 72.27y
ref/alt (all) n=9, 71.78y
alt (all) n=1, 63.48y
ref (female) n=147, 64.69y
ref/alt (female) n=2, 66.50y
ref (all) n=147, 64.69y
ref/alt (all) n=2, 66.50y
ref (male) n=239, 72.59y
ref (female) n=288, 72.02y
ref/alt (male) n=1, 74.50y
ref/alt (female) n=4, 67.86y
ref (all) n=527, 72.28y
ref/alt (all) n=5, 69.18y (172)
ref (male) n=228, 72.70y
ref (female) n=278, 72.00y
ref/alt (male) n=1, 67.27y
ref/alt (female) n=4, 63.43y
ref (all) n=506, 72.31y
ref/alt (all) n=5, 64.20y (90)
ref (female) n=137, 64.75y
ref/alt (female) n=1, 80-85y
ref (all) n=137, 64.75y
ref/alt (all) n=1, 80-85y
ref (male) n=234, 72.42y
ref (female) n=292, 71.96y
ref/alt (male) n=6, 79.31y
ref (all) n=526, 72.17y
ref/alt (all) n=6, 79.31y (849)
ref (female) n=146, 64.80y
ref/alt (female) n=2, 61.00y
ref (all) n=146, 64.80y
ref/alt (all) n=2, 61.00y
reference (male) n=235, 72.67y
reference (female) n=284, 71.97y
homoplasmic (male) n=5, 69.18y (46)
homoplasmic (female) n=6, 73.50y
reference (all) n=519, 72.29y
homoplasmic (all) n=11, 71.54y

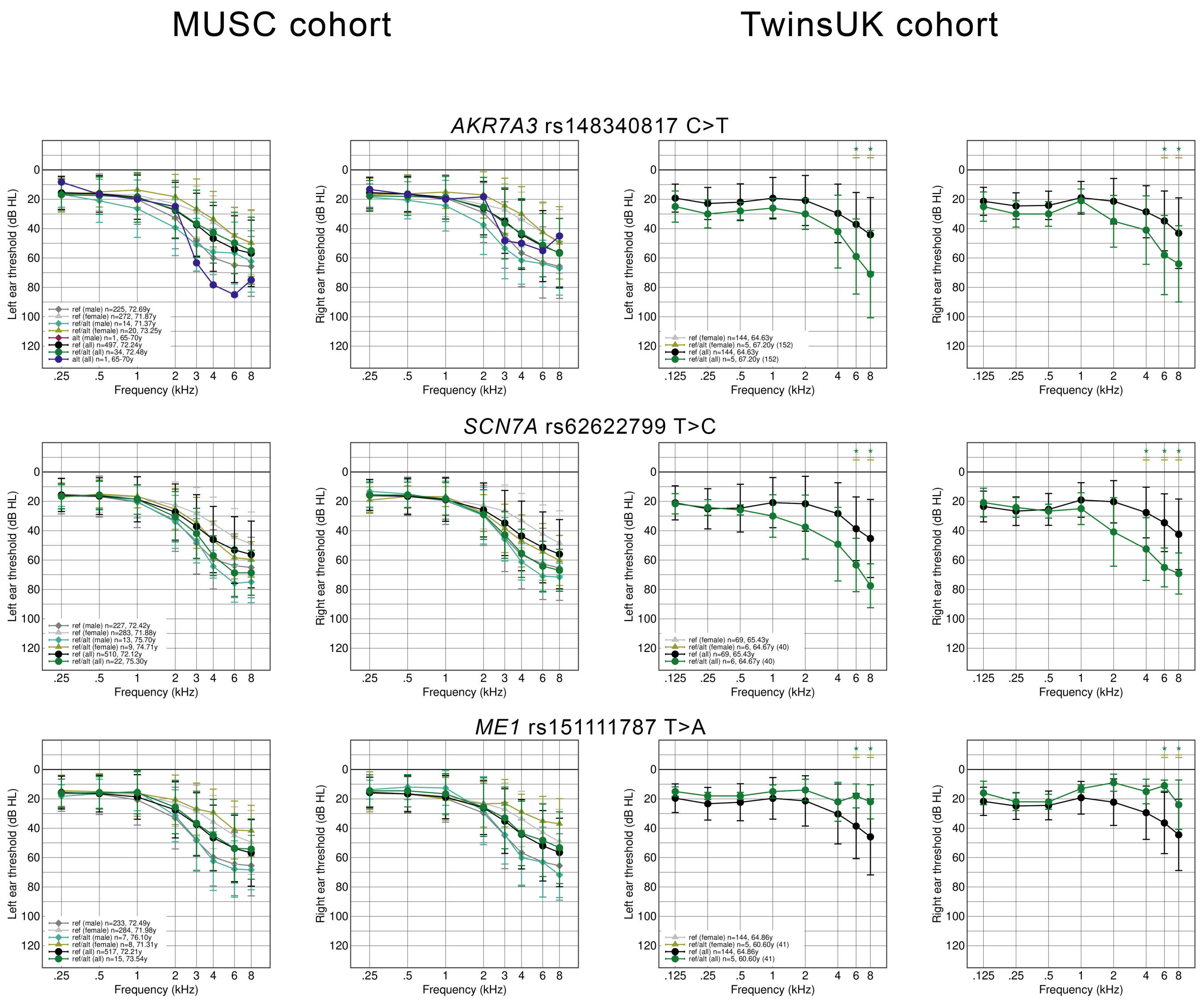
MUSC cohort
TwinsUK cohort
AKR7A3 rs148340817 C>T
SCN7A rs62622799 T>C
ME1 rs151111787 T>A
Left ear threshold (dB HL)
Right ear threshold (dB HL)
Frequency (kHz)
ref (male) n=225, 72.69y
ref (female) n=272, 71.87y
ref/alt (male) n=14, 71.37y
ref/alt (female) n=20, 73.25y
alt (male) n=1, 65-70y
ref (all) n=497, 72.24y
ref/alt (all) n=34, 72.48y
alt (all) n=1, 65-70y
ref (female) n=144, 64.63y
ref/alt (female) n=5, 67.20y (152)
ref (all) n=144, 64.63y
ref/alt (all) n=5, 67.20y (152)
ref (male) n=227, 72.42y
ref (female) n=283, 71.88y
ref/alt (male) n=13, 75.70y
ref/alt (female) n=9, 74.71y
ref (all) n=510, 72.12y
ref/alt (all) n=22, 75.30y
ref (female) n=69, 65.43y
ref/alt (female) n=6, 64.67y (40)
ref (all) n=69, 65.43y
ref/alt (all) n=6, 64.67y (40)
ref (male) n=233, 72.49y
ref (female) n=284, 71.98y
ref/alt (male) n=7, 76.10y
ref/alt (female) n=8, 71.31y
ref (all) n=517, 72.21y
ref/alt (all) n=15, 73.54y
ref (female) n=144, 64.86y
ref/alt (female) n=5, 60.60y (41)
ref (all) n=144, 64.86y
ref/alt (all) n=5, 60.60y (41)

Suppl. Figure 7. Audiograms from the MUSC cohort (left pair) and TwinsUK cohort (right pair) showing threshold differences for carriers of the 41 variants identified in the MUSC cohort and the 4 found in the TwinsUK cohort. The corresponding audiogram from the other cohort is shown where there were carriers of that variant. The numbers in brackets in the keys show the number of permutations giving a similar result for that group. Two audiograms are shown for each variant in each cohort; the thresholds from the left ear are shown on the left, and those from the right ear on the right.
